# Comparative analysis of the end-joining activity of several DNA ligases

**DOI:** 10.1371/journal.pone.0190062

**Published:** 2017-12-28

**Authors:** Robert J. Bauer, Alexander Zhelkovsky, Katharina Bilotti, Laura E. Crowell, Thomas C. Evans, Larry A. McReynolds, Gregory J. S. Lohman

**Affiliations:** Research Division, New England Biolabs, Inc., Ipswich, MA, United States of America; University of Minnesota Twin Cities, UNITED STATES

## Abstract

DNA ligases catalyze the repair of phosphate backbone breaks in DNA, acting with highest activity on breaks in one strand of duplex DNA. Some DNA ligases have also been observed to ligate two DNA fragments with short complementary overhangs or blunt-ended termini. In this study, several wild-type DNA ligases (phage T3, T4, and T7 DNA ligases, *Paramecium bursaria* chlorella virus 1 (PBCV1) DNA ligase, human DNA ligase 3, and *Escherichia coli* DNA ligase) were tested for their ability to ligate DNA fragments with several difficult to ligate end structures (blunt-ended termini, 3′- and 5′- single base overhangs, and 5′-two base overhangs). This analysis revealed that T4 DNA ligase, the most common enzyme utilized for *in vitro* ligation, had its greatest activity on blunt- and 2-base overhangs, and poorest on 5′-single base overhangs. Other ligases had different substrate specificity: T3 DNA ligase ligated only blunt ends well; PBCV1 DNA ligase joined 3′-single base overhangs and 2-base overhangs effectively with little blunt or 5′- single base overhang activity; and human ligase 3 had highest activity on blunt ends and 5′-single base overhangs. There is no correlation of activity among ligases on blunt DNA ends with their activity on single base overhangs. In addition, DNA binding domains (Sso7d, hLig3 zinc finger, and T4 DNA ligase N-terminal domain) were fused to PBCV1 DNA ligase to explore whether modified binding to DNA would lead to greater activity on these difficult to ligate substrates. These engineered ligases showed both an increased binding affinity for DNA and increased activity, but did not alter the relative substrate preferences of PBCV1 DNA ligase, indicating active site structure plays a role in determining substrate preference.

## Introduction

DNA ligases are essential for the *in vivo* maintenance of genome integrity, and have found widespread use in biotechnology applications such as cloning, gene assembly and DNA library assembly. These enzymes catalyze the formation of a phosphodiester bond between adjacent 3′-hydroxyl and 5′-phosphate termini at a DNA break[1, 2]. This DNA joining process follows a ping-pong mechanism, where the first reaction step involves the self-adenylylation of a lysine residue in the ligase active site, generating the catalytically active enzyme form[3]. This catalytically active adenylylated ligase then binds DNA substrate, and the adenylyl group is transferred from the active site lysine onto the 5′-phosphate forming an adenylylated DNA intermediate (AppDNA)[4]. The DNA is then sealed through nucleophilic attack by the adjacent 3′-OH on the 5′-AppDNA intermediate, forming a contiguous phosphodiester backbone[5]. This intermediate is not typically observed in the course of ligation of nicked substrates, but can build up as an abortive side product in difficult to ligate substrates such as those containing mismatched base pairs or gaps between the termini to be ligated[6, 7]. If AppDNA is released from the active site before phosphodiester bond formation, the ligase will rapidly react with ATP, producing an enzyme intermediate that cannot productively bind and react with the free AppDNA intermediate.

In addition to sealing single strand breaks, some DNA ligases also join two DNA fragments with short complementary ssDNA overhangs (cohesive ends) or blunt-ended termini[1, 2, 8, 9]. This activity was first described for bacteriophage T4 DNA ligase, where the sealing of blunt- and cohesive-ended substrates has been extensively studied[10, 11]. However, activity on blunt- and cohesive-ended substrates has since been reported in other DNA ligases, including bacteriophage T3 DNA ligase, *Escherichia coli* DNA Ligase (*E*. *coli* LigA), *Paramecium bursaria* Chlorella virus 1 (PBCV1) DNA ligase, human DNA ligase III (hLig3), human DNA ligase IV (hLIg4), Vaccinia virus DNA ligase, *Mycobacterium smegmatis* LigD, and calf thymus DNA ligases I and II, among others[9, 11–19]. The blunt-end sealing activity for many of these ligases has also been reported to be highly stimulated by the presence of molecular crowding agents such as polyethylene glycol (PEG)[13, 14, 20]. Some of these ligases are thought to be primarily involved in the sealing of nicks between Okazaki fragments during replication (e.g., the phage ligases), while others have been implicated in DNA break repair and non-homologous end joining with (hLig4) or without (hLig3) additional protein factors[19, 21].

**Fig 1:**
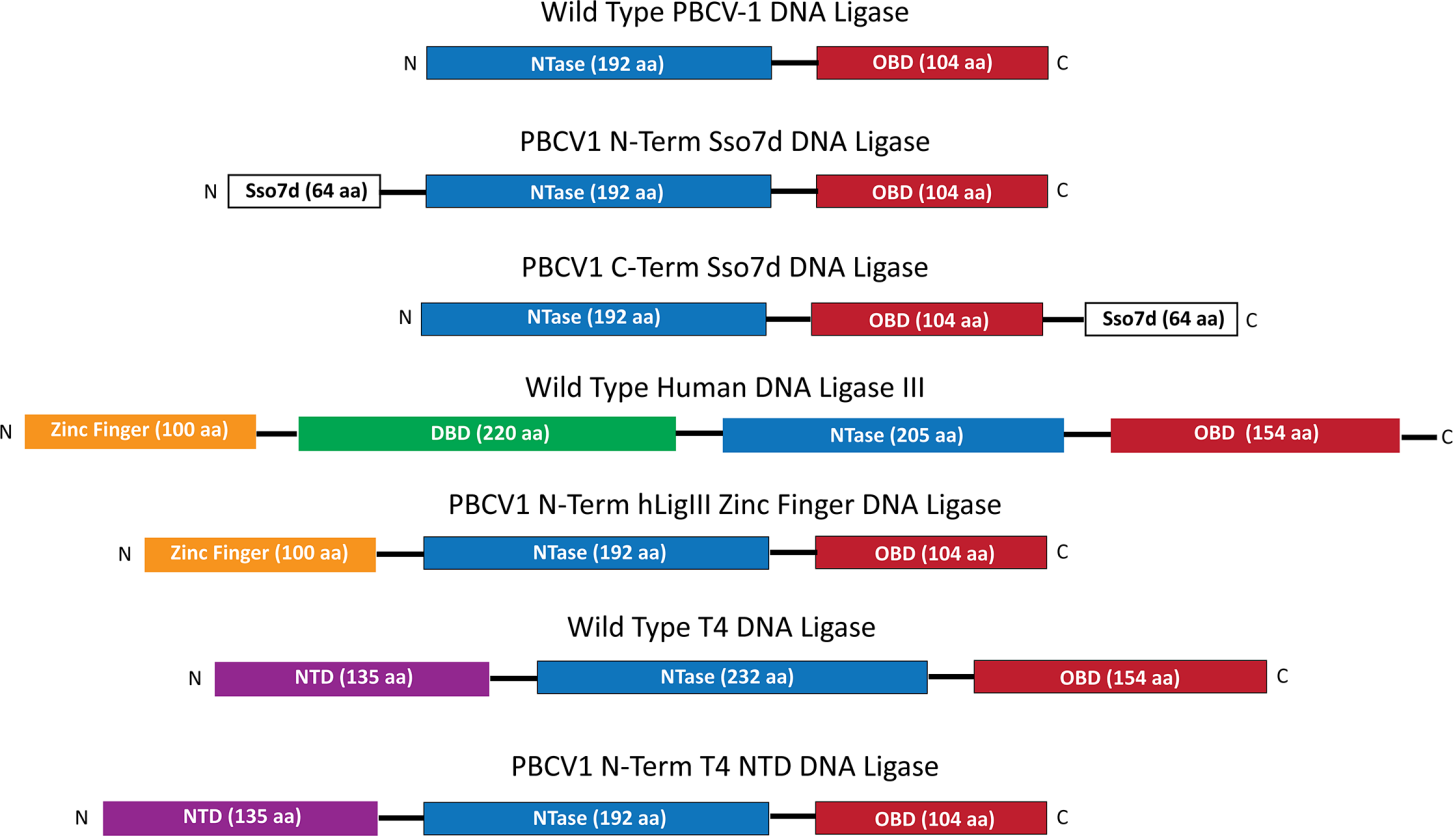
Schematic representation of DNA ligase fusions. All DNA ligases contain a catalytic core NTase domain (blue) and an OBD (red), which are fairly well conserved. Many ligases also have additional domains, such as the N-terminal ZnF (yellow) and DBD (green) in Human Lig3 and the N-terminal domain (NTD) of T4 DNA ligase (purple). Wild type PBCV1 ligase, which contains only the core NTase and OBD domains, was chosen for fusion to other binding domains: Sso7d (white) at both the N- and C-termini, the hLig3 ZnF domain, and the T4 DNA ligase NTD.

Despite a lack of sequence homology between many ligases, their overall domain structure is fairly well conserved (Fig 1)[22]. At minimum, all DNA ligases contain a nucleotidyl transferase domain (NTase), containing the catalytic site, and an oligonucleotide binding domain (OBD) that contains a DNA binding surface and is necessary for enzyme self-adenylylation. Known x-ray structural information shows DNA ligases completely encircling their substrates, and they accomplish this by a third domain[23]. In PBCV1 DNA ligase this third domain is a DNA latch that protrudes from the OB domain and makes contacts with the NTase domain upon encircling its substrate[24]. In other crystalized DNA ligases such as human DNA Ligase I, a third, N-terminal DNA binding domain (DBD) has been observed to play this role[25]. In some cases, blunt/cohesive-end sealing activity has been shown to require specific DNA binding domains present in the ligases. For T4 DNA ligase, it has been shown that removal of residues from the N-terminal domain results in a loss of activity on blunt-ended substrates[26]. In hLig3, it has been proposed that the enzyme utilizes a “jackknife”-like structure when ligating blunt-ended DNA, where its N-terminal poly ADP-ribose polymerase-like zinc finger domain (ZnF) binds one blunt-ended fragment and the catalytic core domain binds the other; this additional ZnF domain has been implicated in its ability to carry out non-homologous end-joining without additional protein factors[9, 19, 27, 28]. In addition, it has been reported that the fusion of DNA binding domains, like the *Sulfolobus solfataricus* small double-stranded DNA binding protein (Sso7d), to T4 DNA ligase increases the enzyme’s activity on blunt/cohesive-ended substrates[29].

The sealing of blunt- and cohesive-ended substrates is commonly used in molecular biology and biotechnology. Examples include the ligation of DNA inserts in plasmids and preparation of libraries for next generation sequencing. These applications almost exclusively call for T4 DNA ligase, which requires high concentrations of enzyme and the presence of PEG for efficient ligation of many difficult-to-ligate ends[11, 20, 29, 30]. Several wild type DNA ligases with known end-joining activity (hLig3, T3 DNA ligase, PBCV-1 DNA ligase, T7 DNA ligase, and *E*. *coli* LigA) were compared to T4 DNA ligase in end-joining reactions with and without PEG[12, 15, 16, 31, 32]. In the current study, we have utilized both ligation of restriction digested λ DNA and ligation of short, defined oligonucleotide substrates to profile the sealing activities of these DNA ligases on several different DNA end structures to evaluate their potential as molecular biology tools. Additionally, PBCV-1 DNA ligase has been fused to several DNA binding domains, including domain swaps from other ligases, and the effect of these fusions on DNA-binding and end-joining activity characterized (Fig 1). Through these studies, the relationship between tight nonspecific DNA-binding and end-joining activity was explored, and insight gained into how catalytic domains and DNA-binding domains are involved in DNA ligase *in vitro* substrate specificity.

## Materials and methods

### General materials

T4 DNA ligase reaction buffer (50 mM Tris-HCl pH 7.5 @ 25°C, 1 mM ATP and 10 mM MgCl_2_) as a 10x stock, NEBNext^®^ Quick Ligation reaction buffer (66 mM Tris pH 7.6 @ 25°C, 10 mM MgCl_2_, 1 mM DTT, 1 mM ATP, 6% Polyethylene glycol (PEG 6000)) as a 5x stock, restriction endonucleases EcoRV, NruI, BstNI, Hpy188I, NdeI, BamHI, as well as λ DNA, 1 M DTT, 6x Purple Loading Dye with SDS, Proteinase K and 10 mM ATP were obtained from New England Biolabs (NEB, Ipswich, MA). Tris-HCl (1 M pH 7.5 @ 25°C) was obtained from Amresco (Solon, OH). Oligonucleotide annealing buffer (10 mM Tris-HCl pH 7.5 @ 25°C, 50 mM KCl and 0.1 mM EDTA) was prepared as a 10X stock. Ligase reaction quench (50 mM EDTA, 0.1% Triton® X-100, 20 U/mL Proteinase K) was prepared as a 1X stock.

### DNA ligases

Bacteriophage T3 DNA ligase, T4 DNA ligase, T7 DNA ligase, Chlorella virus PBCV1 DNA Ligase (sold as SplintR Ligase), and *E*. *coli* LigA were obtained from New England Biolabs (Ipswich, MA).

The human DNA ligase III beta gene was synthesized by Biomatik (Cambridge, Ontario) and subcloned into a pET28 plasmid in frame with an N-terminal His_6_-tag. The gene of the small DNA binding protein (Sso7d) from the hyperthermophilic archaeon *Sulfolobus solfataricus* used in this study was codon optimized for *E*.*coli* expression and synthesized by Celtek Bioscience (Franklin, TN)[33]. The Sso7d gene was fused in frame with the N-terminal His_10_-tag of pET16b and the gene encoding PBCV1 DNA ligase [34], producing PBCV1-Nterm-Sso7d. Sso7d C-terminal fusion to PBCV1 (PBCV1-Cterm-Sso7d) was constructed by ligating the Sso7d gene to the C-terminus of the PBCV1 DNA ligase gene in pET16b with a short 8 amino acid spacer (GTGGGGAV). The sequence encoding the first 119 amino acid residues of hLig3 containing the ZnF was PCR amplified from the wild type gene and fused in frame with the N-terminal His_10_-tag of pET16b and the gene encoding PBCV1 DNA ligase, producing PBCV1-Nterm-ZnF[28, 34]. The sequence encoding the first 135 amino acid residues of T4 DNA ligase containing the presumptive DNA-binding domain (T4NTD) was PCR amplified from the wild type gene. The 5′PCR primer was designed to add an N-terminal His_6_-tag; the resulting PCR product was fused to the N-terminus of PBCV1, producing PBCV1-Nterm-T4NTD. Plasmids containing His_6_-Sso7d-, His_10_-ZnF- and His_6_-T4NTD-binding domains alone were also constructed.

All constructs were expressed in T7 Express *lysY/I*^*q*^ *E*. *coli* cells (NEB) using 0.5 mM IPTG for 2 hours at 30°C for induction. The β isoform of hLig3 was purified as described previously[27]. For all other constructs, after IPTG induction cells were harvested and suspended in 50 mM Tris-HCl buffer pH 7.5, containing 1 M NaCl, 10% glycerol, sonicated and centrifuged at 24000 x g. Cleared extract was applied to Ni-NTA Superflow beads (Qiagen), washed with suspension buffer containing 10 mM imidazole followed by step elution with 50 mM Tris-HCl buffer pH 7.5 containing 10% glycerol, 50 mM NaCl and 100, 300, and 500 mM imidazole for all constructs except 100 mM NaCl for PBCV1-Nterm-ZnF was used. Fractions containing expressed proteins were applied to a HiTrap Heparin column (GE) and eluted with 20 mM Tris-HCl buffer pH 7.5, containing 10% glycerol, 1 mM DTT, 0.5 mM EDTA and a 50 mM– 1 M NaCl gradient. Purified enzymes were dialyzed into a storage buffer containing 10 mM Tris-HCl buffer, pH 7.5 containing 50 mM NaCl, 0.1 mM EDTA, 1 mM DTT, and 50% glycerol and stored at -20°C. For the PBCV1-N-term-ZnF DNA ligase an additional 200 mM NaCl was added to the storage buffer to promote enzyme stability. SDS-Page Gels of purified hLig3, PBCV-1 ligase fusion proteins, Sso7D, and the isolated binding domains can be found in the supporting information (S1 Fig). Nuclease contamination of these proteins was assessed by incubating each protein with an internally labeled ssDNA oligo in T4 DNA ligase reaction buffer and NEBuffer 2 for 1 h at 25°C (See supporting methods, S2 Fig). No degradation of the substrate was observed after 1 hour in either buffer for most proteins; hLig3 showed trace (<5%) degradation.

### DNA substrates

λ DNA was digested as recommended by manufacturer protocols for each restriction enzyme and clean up performed via Monarch^TM^ PCR/DNA cleanup kit (New England Biolabs).

HPLC-purified lyophilized synthetic single-stranded oligonucleotides were obtained from Integrated DNA Technologies (IDT; Coralville, IA) as lyophilized solids. Oligonucleotides were stored as 100 μM stocks in 1X oligonucleotide annealing buffer using mass specifications provided by IDT. All DNA substrates were prepared by combining the 5′-phosphorylated 3′-6-carboxyfluorescein (FAM)-labeled fragment with the unlabeled or carboxy-X-rhodamine (ROX)-labeled complementary strand in a 1:1.1 molar ratio in DNA annealing buffer (**Table 1**). This mixture was heated to 95°C and cooled to room temperature slowly over at least two hours. The concentration of the annealed DNA stock is expressed in terms of the FAM-labeled fragment. All DNA constructs have one terminal 5′ PO_4_ (on the end opposite to the FAM label) to allow for ligation.

**Table 1:** DNA substrates. Substrates are shown aligned as annealed. A boldface and underlined nucleotide indicates a 3’-fluorescent label; 3′**C** is labeled with FAM, 3′**A** with ROX. SBO substrates combine both the FAM and ROX fragments in a single reaction.

**A/T Blunt**	5′–pATCTGGGACCTACAATGTACCAGAAGCGT**C**–3′ 3′–TAGACCCTGGATGTTACATGGTCTTCGCAG–5′
**G/C Blunt**	5′–pCGATGGGACCTACAATGTACCAGAAGCGT**C**-3′ 3′–GCTACCCTGGATGTTACATGGTCTTCGCAG–5′
**5′SBO A/T** **FAM-fragment**	5′–pTGGAGGGACCTACAATGTACCAGAAGCGT**C**-3′ 3′–CCTCCCTGGATGTTACATGGTCTTCGCAG–5′
**5′SBO A/T** **ROX-fragment**	5′–p–AGGAGGAGAAGTAAAGTTAT**A**–3′ 3′–CCTCCTCTTCATTTCAATAT-5′
**5′SBO G/C** **FAM-fragment**	5′-pGGGAGGGACCTACAATGTACCAGAACGCT**C**-3′ 3′-CCTCCCTGGATGTTACATGGTCTTCGCAG-5′
**5′SBO G/C** **ROX-fragment**	5′–pCGGAGGAGAAGTAAAGTTAT**A**–3′ 3′–CCTCCTCTTCATTTCAATAT-5′
**3′SBO A/T** **FAM-fragment**	5′–pGATGGGACCTACAATGTACCAGAAGCGT**C**–3′ 3′-ACTACCCTGGATGTTACATGGTCTTCGCAG-5′
**3′SBO A/T** **ROX-fragment**	5′-pGACATAGAAGTAAAGTTAT**A**-3′ 3′-TCTGTATCTTCATTTCAATAT-5′
**3′SBO G/C** **FAM-fragment**	5′-pGATGGGACCTACAATGTACCAGAAGCGT**C**-3′ 3′-CCTACCCTGGATGTTACATGGTCTTCGCAG-5′
**3′SBO G/C** **ROX-fragment**	5′-pGACATAGAAGTAAAGTTAT**A**-3′ 3′-GCTGTATCTTCATTTCAATAT-5′
**2BO**	5′–pTATGGGGACCTACAATGTACCAGAAGCGT**C**–3′ 3′–ACCCCTGGATGTTACATGGTCTTCGCAG–5′
**4BO**	5′–pGATCCTTAGATAGTATACTGAGTTCTGTAAACGAGCTATTGAATT**C**–3′ 3′–GAATCTATCATATGACTCAAGACATTTGCTCGATAACTTAAG–5′
**Anisotropy 24mer dsDNA**	5′-GATCGTCCTTGGTGATCATGCAT**C**-3′ 3′-CTAGCAGGAACCACTAGTACGTAG-5′

### Blunt/cohesive ended oligo ligation assay

Standard ligation assay mixtures were composed of 1X T4 reaction buffer or NEBNext^®^ Quick Ligation reaction buffer, 100–1000 nM ligase, and 100 nM FAM-labeled DNA substrate as indicated in each figure legend, in a reaction volume of 20 μL. Reactions were performed at 25°C for all DNA ligases. Components were gently mixed by pipetting and incubated at reaction temperature for 5 minutes prior to initiation by the addition of the DNA substrate. Reactions were quenched by a 1:1 vol:vol addition of ligase reaction quench at times as indicated in each figure legend and incubated 30 min at 37°C to allow for ligase cleavage by proteinase K. The ligated products were analyzed by capillary gel electrophoresis as described previously[5, 35–37]. Reported values are the average of a minimum of three replicates, with the errors the standard deviation of the measurements.

Reaction endpoints and protein dsDNA nuclease contamination level were assessed via an overnight (18 h) incubation of each substrate (100 nM) with 1000 nM of each ligase at 25°C in NEBNext^®^ Quick Ligation reaction buffer. These results can be found in the Supplemental Information (S3 and S4 Figs).

### Blunt/cohesive ended λ DNA digest ligation assay

Standard λ DNA digest ligation assay mixtures included 7 μM ligase in T4 DNA ligase reaction buffer or Quick Ligation reaction buffer and 0.5 μg of the digested DNA in a 10 μL total reaction volume. Reactions were performed at 25°C for all DNA ligases with a 1 hour incubation time. After ligation, 1 μL of proteinase K was added to the reaction along with purple loading dye with SDS and the reaction mixture digested for 30 minutes at 37°C to remove any bound ligase from the sealed DNA. The ligated DNA was then separated by gel electrophoresis using a 1% agarose gel containing ethidium bromide for visualization. Each λ DNA digest ligation assay was repeated at least twice using independent digestions of λ DNA with each restriction enzyme; a representative gel is shown in the text. Degree of ligation was not quantified; gels are used only to assign a qualitative degree of ligation under the conditions described in the text and were in general agreement between replicates.

### Ligase-DNA fluorescence anisotropy binding experiments

Anisotropy assays were performed in T4 DNA ligase reaction buffer. Ligases were bound to a 24mer dsDNA substrate (Anisotropy 24mer dsDNA, Table 1) where a 3′ FAM was present on one strand. Reaction substrate concentration ranged from 750 pM– 4 nM as indicated in each figure legend. Varying concentrations of each ligase were titrated into constant amounts of substrate as described in each figure legend. Anisotropy values were obtained using a Fluoromax-4 fluorimeter with automated polarizers (HORIBA Jobin Yvon, Edison NJ) using an excitation wavelength of 492 nm. Anisotropy measurements taken at an emission wavelength maximum of 517 nm, for one second integration times. All data points from each experimental replicate are the average of 10 consecutive readings, and reported data represent the average of at least three independent titrations. Error bars on data points represent the standard error of the titration replicates.

Fluorescence anisotropy is calculated using the following equation:

$$r=\frac{I_{VV}-{GI}_{VH}}{I_{vv}+2\;I_{VH}}$$ (Eq 1)

Where *I* is the polarization intensity and the subscripts, *V* and *H* represent vertical or horizontally polarized light. *G* is a correction factor for any differences in the intensity of horizontally and vertically polarized light intrinsic to the instrument and is calculated automatically by the included fluorescence software.

The change in anisotropy (Δr) was fit either to a single binding equation:

$$\Delta r=\frac{A*[E]}{K_{d}+[E]}$$ (Eq 2)

Where *A* is the reaction amplitude and *K*_*d*_ is the dissociation constant for the interaction between the ligase and DNA, or to the Hill equation for cooperative binding:

$$\Delta r=\frac{A*{\left[E\right]}^{n}}{{K_{d}}^{n}+{\left[E\right]}^{n}}$$ (Eq 3)

Where *A* is the reaction amplitude, *K*_*d*_ is the dissociation constant for the interaction between the ligase and DNA and *n* is the Hill coefficient. All non-linear least squares regression analysis was performed using KaleidaGraph (Synergy Software, V 4.5.1). Equations were fit to the average of three experimental replicates to determine *K*_d_; the reported is the error in this parameter as determined by the regression analysis.

## Results and discussion

### DNA ligase activity on restriction enzyme digested λ DNA

The blunt/cohesive-end sealing activity of wild type ligases including T4 DNA ligase, T3 DNA ligase, PBCV1 DNA ligase, and hLig3 were initially profiled through use of a λ DNA restriction digest ligation assay. This assay involved λ DNA that had been digested by a variety of restriction enzymes to produce DNA fragments containing specific end structures. The restriction enzymes used were EcoRV (Blunt End A/T), NruI (Blunt End G/C), BstNI (5′ single base overhang A/T), Hpy188I (3′ single base overhang A/T or G/C), NdeI (2 base overhang AT/TA), and BamHI (4 base overhang GATC/CTAG). The digested DNA (50 pg/μL) was then ligated by reaction with a high concentration (7 μM) of one of the above listed ligases and one of two reaction buffers (NEB’s T4 Ligase reaction buffer and NEBNext^®^ Quick Ligation reaction buffer). The major difference between the two buffers is the inclusion of the crowding agent PEG-6000 (6%) in NEBNext^®^ Quick Ligation reaction buffer.

**Fig 2:**
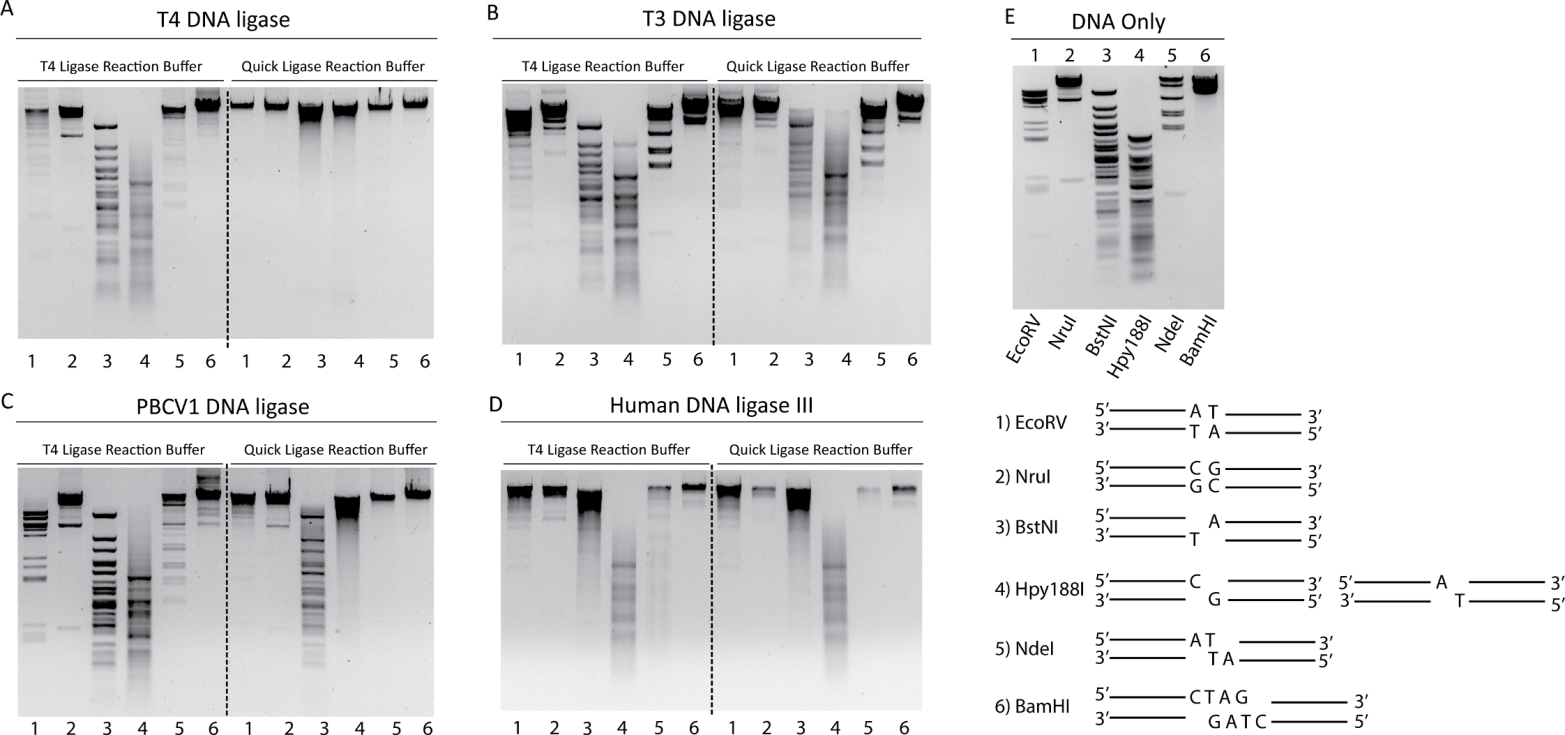
Wild type DNA ligase λ DNA digest ligation assay. Agarose gel electrophoresis of λ DNA cut by EcoRV (A/T Blunt, **1**), NruI (G/C Blunt, **2**), BstNI (5′ SBO, **3**), Hpy188I (3′SBO, **4**), NdeI (2 BO, **5**) and BamHI (4 BO, **6**), generating DNA fragments with ligatable ends. 0.5 ng of the cut DNA was ligated in the presence of T4 ligase reaction buffer (50 mM Tris-HCl pH 7.5 @ 25°C, 1 mM ATP and 10 mM MgCl_2_) or NEBNext^®^ Quick Ligation reaction buffer (66 mM Tris pH 7.6 @ 25°C, 10 mM MgCl2, 1 mM DTT, 1 mM ATP, 6% polyethylene glycol (PEG 6000)) and 7 μM of the indicated DNA ligase for 1 hour at 25°C. Ligation assays performed with T4 DNA ligase (A), T3 DNA ligase (B), PBCV1 DNA ligase (C) and, hLig3 (D), respectively. E) Gel of restriction enzyme digested λ DNA samples as well as a schematic depiction of each substrate. The DNA fragments were visualized using ethidium bromide stain.

λ DNA digest ligation revealed that PEG stimulates all the tested wild type ligases, with T4 DNA ligase showing the best activity on all of the end types (Fig 2). The remaining ligases all show activity on the two blunt-ended substrates, especially in the presence of PEG. T3 DNA ligase did not have significant activity on substrates with a single- or two-base overhang, even in PEG containing buffer (Fig 2B). However, PBCV1 DNA ligase showed activity on single base overhang substrates (SBO), displaying a preference for 3′SBO over 5′SBO while hLig3 showed a preference for 5′SBO over 3′SBO, and both showed the ability to seal the two- and four-base overhang cohesive ends (Fig 2C and 2D). It should be noted that hLig3 remained very tightly bound to the ligated DNA after reaction, necessitating the inclusion of a 30-minute proteinase K treatment in the presence of SDS to prevent gel shifts.

### Profiling blunt/cohesive end ligation by capillary electrophoresis

In order to characterize the efficiency of these enzymes in a more quantitative fashion, a ligation assay involving short fluorescently (FAM or ROX) labeled DNA oligo substrates with blunt and cohesive ends (blunt, 5′ single-base overhang (5′SBO), 3′ single-base overhang (3′SBO), 2-base overhang (2BO), and 4-base overhang (4BO)) was utilized. Two blunt-ended substrates were designed such that the base pairs were either A/T (**Table 1, A/T Blunt**) or G/C (**Table 1, G/C Blunt**) at the ligation junction. Two pairs of each of 3′SBO substrates were designed, differing only in that the overhang base pair was either an A/T or G/C; likewise, pairs of 5′SBO substrates were designed with A/T or G/C base pairs at the overhang. The 2BO substrate was modeled on the NdeI cut-site with the overhanging bases being 5′AT; the 4BO substrate was modeled on the BamHI cut-site and contains an overhang with the sequence 5′GATC.

**Fig 3:**
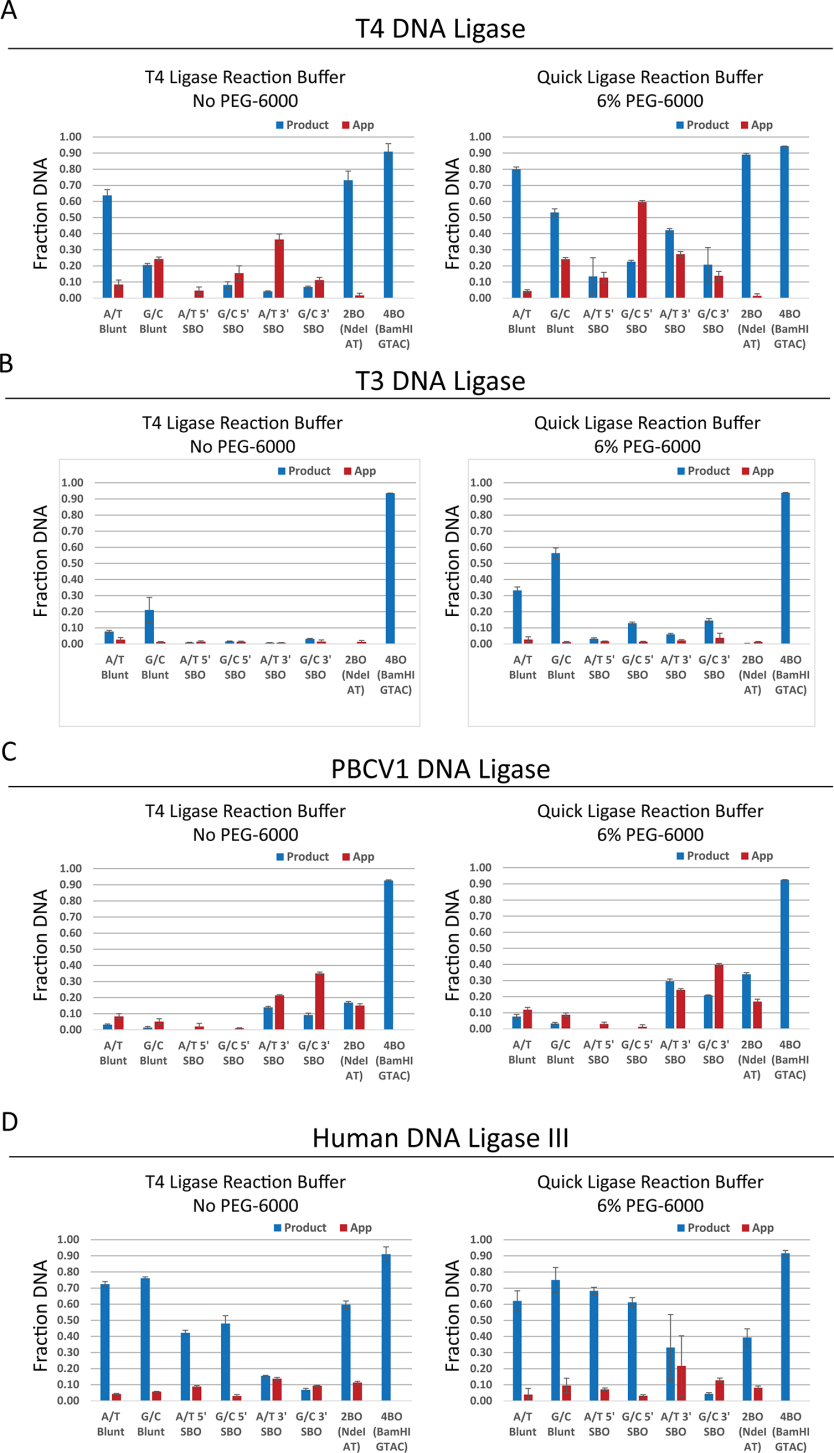
Wild type DNA ligase blunt/cohesive capillary electrophoresis assay. Bar graphs depict the fraction of either ligated DNA (product, blue) or abortive adenylylation (App, red) produced in a 20-minute sealing reaction with the indicated DNA substrate. Reactions included 1 μM of the DNA ligase, 100 nM of the substrate and reaction conditions consisting of either T4 DNA ligase reaction buffer (50 mM Tris-HCl pH 7.5 @ 25°C, 1 mM ATP and 10 mM MgCl_2_) or NEBNext^®^ Quick Ligation reaction buffer (66 mM Tris pH 7.6 @ 25°C, 10 mM MgCl_2_, 1 mM DTT, 1 mM ATP, 6% Polyethylene glycol (PEG 6000)). Ligation assays performed with T4 DNA ligase (A), T3 DNA ligase (B), PBCV1 DNA ligase (C) and hLig3 (D), respectively Experiments were performed in triplicate; the plotted value is the average and the error bars represent the standard deviation across replicates.

A Capillary gel electrophoresis-based approach was used to monitor reaction progress and allowed for quantitation of both adenylylated DNA intermediate and sealed product formation. We examined a variety of wild type ligases, including T3 DNA ligase, T4 DNA ligase, T7 DNA ligase, PBCV1 DNA ligase, hLig3, and *E*. *coli* LigA. The two ligases with the highest activity on the blunt/cohesive panel substrates were T4 DNA ligase and hLig3 (Fig 3A and 3D) both of which ligated blunt-ended substrates well with T4 DNA ligase sealing over 60% of the A/T Blunt substrate in the presence or absence of PEG, and 50% of the G/C blunt substrate in the presence of PEG. Human Lig3 had similar activity on both blunt-ended substrates in the presence and absence of PEG forming ~70% sealed product. Both T4 DNA ligase and hLig3 had the highest activity on SBO substrates in the presence of PEG; however, the preference for the type of SBO (3′ vs 5′) was different. T4 DNA ligase formed more products on 3′SBO substrates with a bias towards A/T paired ends. For hLig3 the inverse was true, as the enzyme sealed 5′SBO substrates well and with no observable bias for A/T or G/C paired ends. T4 DNA ligase showed a preference for blunt ends over either SBO, with the lowest activity on 5′SBO, while hLig3 has equal ability to ligate blunt ends and 5′SBO, with much lower activity on 3′SBO.

T3 DNA ligase, for instance, had limited activity on all but 4BO substrates in the absence of PEG (Fig 3B), while in the presence of PEG, it was able to ligate blunt-ended substrates almost as well as either T4 or hLig3. PBCV1 DNA ligase (Fig 3C) did not show significant activity on blunt-ended or 5′SBO substrates but does show activity on 3′SBO and 4BO substrates, with activity comparable to or better than T4 DNA ligase in the absence of PEG and comparable to T4 DNA ligase in the presence of PEG. The other tested DNA ligases (*E*. *coli* LigA, T7) did not display significant activity on the blunt/cohesive substrate panel outside sealing of the 4BO (S1 Table, Supporting Information). Thus, ligases differ not only in their absolute activity in end-joining, but in which end types are relatively preferred for each ligase. These differences in substrate preference indicate that the ease of ligation is not an intrinsic property of the DNA structures or overhang annealing, which would lead to the same order of substrate preference for each ligase, but related to the structure of the DNA ligases, with each ligase better able to accommodate some end types over others.

Interestingly, not all ligases behaved the same with respect to the degree of AppDNA intermediate build-up. AppDNA was quite prevalent in reactions by T4 DNA ligase and PBCV1 DNA ligase. In some cases, e.g., T4 DNA ligase and 5′SBO and PBCV1 with 3′SBO, the amount of AppDNA observed exceeded the amount of ligated product. This result suggests that the ligation reaction was failing due to dissociation of the ligase from the substrate after adenylyl transfer but before phosphodiester bond formation. Conversely, T3 DNA ligase and hLig3 showed very little AppDNA, irrespective of whether a substrate was one easily or poorly ligated by the ligase. The precise mechanistic underpinnings of this result are unclear from the current work, and will require further study to determine. One possible explanation is that adenylyl transfer was almost always followed by rapid phosphodiester bond formation. Alternatively, the ligase formed a very tight complex with AppDNA, such that it seldom dissociated from the enzyme active site before ligating to a suitable partner. A final possibility is that, for these enzymes, self-adenylylation is not rapid and released AppDNA could be more easily bound and reacted by deadenylylated ligase.

End-joining ligation by these ligases was also examined in the presence of 150 mM NaCl. It is noteworthy that increasing the monovalent cation concentration to near physiological level eliminated nearly all end-joining ability for all ligases (S5 Fig, Supporting Information), with good activity retained only on the 4BO substrate. This result held true even for T3 DNA ligase, which was previously reported to be quite salt tolerant in the presence of 15–20% PEG[16]. In the current study, T3 ligase had only trace blunt ligation activity even in the presence of 6% PEG, while PBCV1 DNA ligase retained some activity on 3’-SBO and 2BO, and T4 DNA ligase had low activity on A/T blunt and 2BO substrates. Only hLig3 retained some activity on all substrates in the presence of PEG, though greatly reduced in comparison to the low-salt experiments. This result indicates that the end-joining activity is greatly reduced under physiologically relevant ionic strengths, at least in the absence of required additional factors to localize the ligase to the DNA and/or hold the ends in close proximity[19, 21, 38, 39]. The higher degrees of molecular crowding present *in vivo* as compared to these *in* vitro experiments may also overcome this inhibition effect.

### Fusion of DNA-binding domains increased end-joining activity of PBCV1 DNA ligase but did not alter relative substrate preferences

It was previously reported for T4 DNA ligase that fusing DNA binding domains to the ligase increased its activity on blunt/cohesive-ended substrates[29]. Here, it was tested whether this was also true for PBCV1 DNA ligase, which lacks any significant N-terminal DBD, having naturally only an NT domain and an OB domain, with a tiny “latch” domain extending out of the OB domain (Fig 1). We hypothesized that this ligase should be amenable to N-terminal fusions, and investigated both whether simply increasing DNA affinity would increase end-joining activity and whether transferring N-terminal binding domains from other ligases could transfer the activity and/or substrate preferences of other ligases to PBCV-1. Several different fusions were thus created. N- and C-terminal fusions of the *S*. *solfataricus* small DNA binding protein (Sso7d), as well as N-terminal fusions of the ZnF domain of hLig3, and the N-terminal putative DBD domain of T4 DNA ligase. (Fig 2)[9, 26–28, 40]. In this study, only the first 119 aa of the wild type hLig3 sequence were used, identified as a minimal ZnF fold based on homology to the ZnF of poly ADP-ribose polymerase, not the remainder of the previously identified pre-DBD sequence (residues 120–170)[28]. Both the λ DNA digest ligation assay, as well as the CE panel, were used to profile the effect of the extra binding domain on the blunt/cohesive-end sealing activity of the ligase.

**Fig 4:**
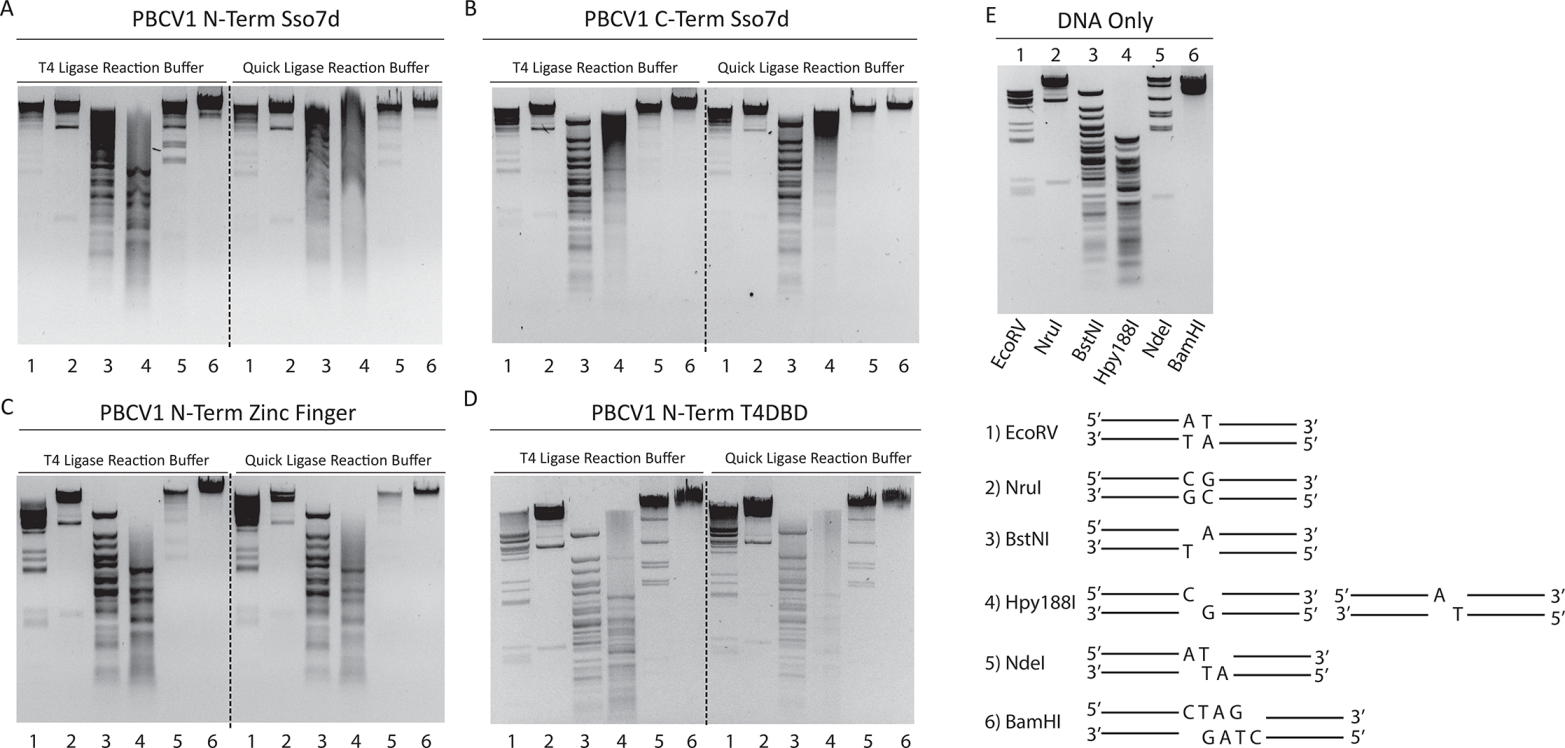
Effect of DBDs on blunt/cohesive end λ DNA Re-ligation. Agarose gel electrophoresis of λ DNA cut by EcoRV (A/T Blunt, 1), NruI (G/C Blunt, 2), BstNI (5′ SBO, 3), Hpy188I (3′SBO, 4), NdeI (2 BO, 5) and BamHI (4 BO, 6), generating DNA fragments with ligatable ends. 0.5 ng of the cut DNA was ligated in T4 ligase reaction buffer (50 mM Tris-HCl pH 7.5 @ 25°C, 1 mM ATP and 10 mM MgCl_2_) or NEBNext^®^ Quick Ligation reaction buffer (66 mM Tris pH 7.6 @ 25°C, 10 mM MgCl_2_, 1 mM DTT, 1 mM ATP, 6% Polyethylene glycol (PEG 6000)) and 7 μM of the indicated DNA ligase for 1 hour at 25°C. Ligation assays performed with PBCV1-Nterm-Sso7d (A), PBCV1-Cterm-Sso7d terminus (B), PBCV1-Nterm-ZnF (C), PBCV1-Nterm-T4NTD (D). (E) Gel of restriction enzyme digested λ DNA samples as well as a schematic depiction of each substrate. The DNA fragments were visualized using ethidium bromide stain.

**Fig 5:**
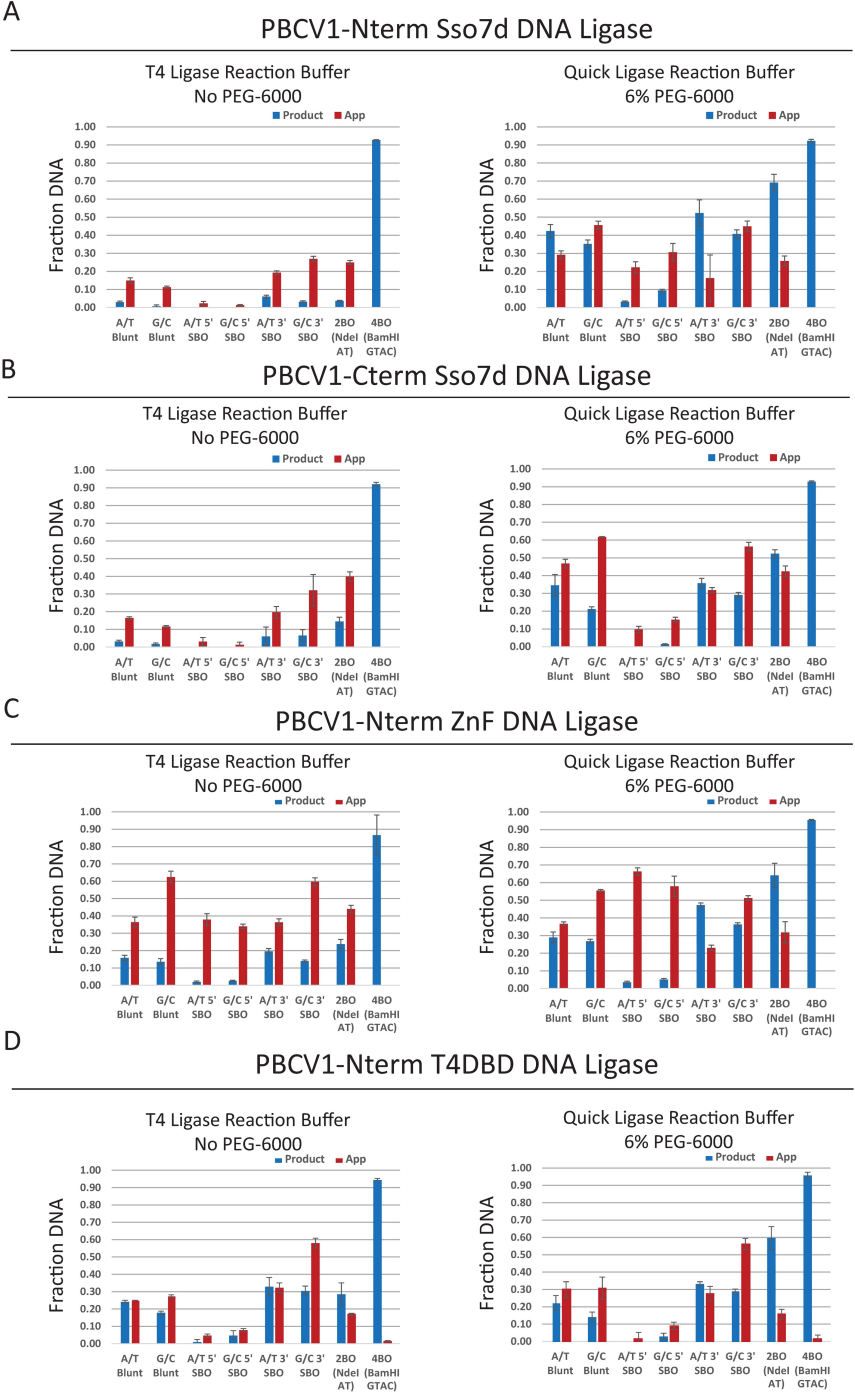
Effect of DBD on blunt/cohesive end ligation. Bar graphs depict the fraction of either ligated DNA (product, blue) or abortive adenylylation (App, red) produced in a 20-minute sealing reaction with the indicated DNA substrate. Reactions included 1 μM of the DNA ligase, 100 nM of the substrate and reaction conditions consisting of either T4 DNA ligase reaction buffer (50 mM Tris-HCl pH 7.5 @ 25°C, 1 mM ATP and 10 mM MgCl_2_) or NEBNext^®^ Quick Ligation reaction buffer (66 mM Tris pH 7.6 @ 25°C, 10 mM MgCl_2_, 1 mM DTT, 1 mM ATP, 6% Polyethylene glycol (PEG 6000)). Ligation assays performed with PBCV1-Nterm-Sso7d (A), PBCV1-Cterm-Sso7d terminus (B), PBCV1-Nterm-ZnF (C), PBCV1-Nterm-T4NTD (D). Experiments were performed in triplicate; the plotted value is the average and the error bars represent the standard deviation across replicates.

Previous reports on fusing the Sso7D domain to T4 DNA ligase showed a ~2-fold increase in activity on blunt ligation[29]. For the PBCV-1 DNA ligase- Sso7D fusions, it was observed in the λ digest ligation assay that both N- and C-terminal Sso7d fusions likewise stimulated the activity of the ligase over that of the wild type enzyme and that the N-terminal Sso7d fusion had better activity on 5′SBO substrates than did the C-terminal fusion (Fig 4A and 4B, compare to Fig 2C). These results are confirmed by the oligo DNA ligation assay, where a significant stimulation of enzyme on blunt-ended and 3′SBO substrates was observed for the Sso7d fusion ligases, with increases in both ligation product and AppDNA intermediate observed (Fig 5A and 5B, compare to Fig 3C). As in the case for the wild-type enzymes, PEG stimulated ligation yields significantly in the oligo ligation assay. In the presence of PEG, we observed a ~2-fold increase in yield for 3′SBO and 2BO ligation, similar to the stimulation this fusion was reported to generate in T4 DNA ligase. However, blunt and 5′SBO showed significantly more stimulation, ~5-fold increase compared to wild type. As a control, these reactions were repeated with wild type PBCV-1 and the Sso7d domain in trans; no enhancement to yield was observed in this case (S6A Fig, Supporting Information).

The fusion of N-terminal binding domains from other DNA ligases gave a very similar effect to fusing Sso7d. While the λ DNA ligation assay did not seem to show significant stimulation for the ZnF fusion ligase over wild type (Figs 2C and 4C), the oligo ligation assay showed that this domain stimulates the activity of PBCV1 DNA ligase even in the absence of PEG on blunt-ended and 3′ SBO substrates, and this stimulation is increased by the presence of PEG (Figs 3C and 5C). The observed product yields increased to a degree similar to that seen for the N-terminal Sso7d fusion. The ZnF fusion also displayed increases in the amount of AppDNA intermediate observed, especially dramatically for the substrates for which the wild-type enzyme was least active (5′SBO). As with Sso7d, including the ZnF domain in trans with wild type PBCV-1 DNA ligase resulted in no enhancement (S6B Fig, Supporting Information).

Finally, the fusion of the N-terminal domain of T4 DNA ligase did not seem to show stimulated activity over the wild-type enzyme in the λ DNA digest ligation assay (Figs 2C and 4D). However, the oligo ligation assay showed fusion of the T4 N-terminal domain increased product and AppDNA intermediate yields for blunt, 3′SBO, and 2BO substrates by ~2-fold, both in the presence and absence of PEG (Figs 3C and 5D). The degree of increase was similar to the increases in both product formation and abortive ligation seen for the other PBCV-1 DNA ligase fusions. Interestingly, the dramatic increase in AppDNA intermediate observed for the ZnF fusion with the 5′SBO substrates was not observed for the T4-NTD fusion. Ultimately, the 5′SBO substrate remained the least preferable. In this case, the T4 NTD alone was not stable in solution, thus the in trans control was not performed.

Overall, we observed that DNA binding domain fusions greatly increased the chance of successful adenylyl group transfer, even when yield increases were modest or negligible. The likely mechanism of increased yield and AppDNA is through increasing affinity for the substrate, though in cases where AppDNA dominated, the ligase fusions were not necessarily able to remain bound long enough to complete ligation or, potentially, to properly orient a second DNA in the 3′-hydroxyl position to allow phosphodiester bond formation.

Interestingly, regardless of the binding domain fused, PBCV1 retained preference for 3′SBO substrates over all others and did not, for example, gain the hLig3 preference for 5′SBO when fused to the hLig3 ZnF. These results support the idea that blunt/cohesive-end ligation ability is intrinsic to the ligase active site, and that each ligase has a unique profile of activity on these substrates. That is, increasing DNA binding affinity for a given ligase can increase its overall activity, but the relative substrate preferences are controlled by the wild type ligase active site structure, or the overall DNA binding pocket formed by the NT and OB domains.

### Quantification of ligase and ligase fusion DNA binding by fluorescence anisotropy

Fluorescence anisotropy-based binding titrations were used to determine the dissociation constants for the most active DNA ligases tested in this study. Initially, PBCV1 DNA ligase, T4 DNA Ligase, hLig3, and T3 DNA ligase were characterized for their nonspecific DNA binding affinity using a 24mer 3′-FAM labeled, non-ligatable DNA (Table 2, S7 Fig) in T4 ligase reaction buffer conditions (50 mM Tris-HCl pH 7.5, 1 mM ATP and 10 mM MgCl_2_). T3, T4 and PBCV1 DNA ligases all had μM affinities for DNA, with T3 being the weakest binder. The mammalian hLig3, by contrast, displayed very tight binding (*K*_d_ = 3.8 ± 0.2 nM), 500-fold tighter than T4 DNA ligase. It is clear that overall affinity to nonspecific DNA does not correlate with activity–notably, the two ligases most active in overall end-joining efficacy, hLig3 and T4 DNA ligase, were at opposite extremes of binding affinity for DNA. This result suggests that tight nonspecific DNA binding is one mechanistic strategy for high efficiency end-joining ligation, but not the only strategy.

**Table 2:** Measured binding constants.

Enzyme	*K*_d_ (DNA 24-mer)^a^	Blunt Ligation Yield^b^
**T3 DNA Ligase**	6400 ± 400 nM	33 ± 2%
**T4 DNA Ligase**	1900 ± 100 nM	41 ± 1%
**PBCV1 DNA Ligase**	990 ± 80 nM	8 ± 1%
**hLig3**	3.8 ± 0.2 nM	62 ± 6%
**PBCV1-Nterm-Sso7d**	7.7 ± 0.5 nM	43 ± 4%
**PBCV1-Cterm-Sso7d**	29 ± 2 nM	35 ± 6%
**PBCV1-NTerm-ZnF**	20 ± 3 nM	29 ± 3%
**PBCV1-Nterm-T4NTD**	243 ± 39 nM	22 ± 4%
**Sso7d**	430 ± 60 nM	N/A^c^
**hLig3 ZnF**	21 ± 1 nM	N/A

a–Determined through fluorescence anisotropy, see S7 Fig (Supporting Information) for binding data and fits. Reported values are the average of a minimum of three independent titrations with the uncertainty the standard error across replicates.

b–Ligation yield as quantitated by capillary gel electrophoresis, 1 μM of the DNA ligase, 100 nM A/T Blunt substrate, NEBNext^®^ Quick Ligation reaction 20 min at 25C. Data from Figs 3 and 5 and S1 Table (Supporting Information).

c–Not applicable.

As controls, the DNA binding affinity was also determined for the Sso7d and hLig3 ZnF binding domains alone; the binding affinity of the T4 NTD alone could not be directly quantified by anisotropy due to precipitation of this isolated domain during analysis. The *K*_d_ of Sso7d was determined to be 430 ± 60 nM in T4 DNA ligase assay buffer with the 24-mer anisotropy DNA substrate, falling within the range that has been reported for this interaction (~120 nm– 10 μM, depending in buffer conditions and substrate sequence)[41, 42]. The *K*_d_ for the hLig3 ZnF was determined to be 21 ± 1 nM, suggesting this domain was the source of much of the tight DNA binding affinity of hLig3. Previous studies found considerably weaker *K*d for the binding of the isolated ZnFdomain to DNA; however different buffer conditions (higher monovalent cation and no magnesium), and use of a domain that was 170 aa rather than the 119 aa domain used in this study, make meaningful comparison to this prior experiment difficult[28].

DNA binding affinity measured for all PBCV1 ligase-binding domain fusion constructs was significantly increased, even when the isolated protein (Sso7d) or source ligase (T4) were relatively weak binding (Table 2). With a Sso7d fused to the N-terminus of PBCV1 DNA ligase, 130-fold tighter DNA binding (*K*_d_ = 7.7 ± 0.5 nM) was observed when compared to the wild type enzyme; the C-terminal fusion increased the binding affinity 34-fold. The PBCV1-N-term ZnF construct had a 50-fold tighter binding affinity for DNA than the wild type enzyme (*K*_d_ = 20 ± 3 nM), demonstrating that the tight affinity this domain gives hLig3 to DNA can be transferred to another ligase. Finally, the construct fusing the N-terminal domain of T4 ligase on to PBCV1 DNA ligase increased the affinity of the ligase for DNA by ~4 fold (*K*_d_ = 243 ± 39 nM), indicating that this domain indeed is involved in DNA binding[26, 28]. Interestingly the fusion of Sso7d resulted in a binding affinity tighter than either the protein alone; this strong avidity effect is likely the result of cooperative binding between the domains. A similar effect was observed in prior studies involving the fusion of this protein to polymerases[43]. Conversely, the binding of the ZnF fusion was approximately the same as the tight binding ZnF domain alone, suggesting this domain dominates the interaction with DNA, and that the ligase binding domain cannot bind cooperatively with the ZnF in this construct.

While the strength of DNA binding does not correlate well with activity of different wild type ligases, the increase in affinity for nonspecific DNA binding across the PBCV-1 DNA ligase fusions does appear to correlate well with the increase in activity observed, with the tighter-binding fusions correspondingly showing higher activity, and the weakest binding (T4 NTD fusion) showing a gain in AppDNA production but not ligated product. This result again indicates tight nonspecific binding of DNA can lead to high end-joining activity; however, ligases such as T3 and T4 DNA ligase appear to accomplish efficient end-joining without this high nonspecific binding affinity.

## Conclusion

Elucidation of the end-joining substrate preferences of DNA ligases opens up the possibility of their selective use when the ligation of a particular end is desired over others. For example, while T4 and hLig3 efficiently ligate many different junctions in the presence of PEG, selective ligation of blunt ends or 3′SBO could be achieved by using T3 or PBCV1 DNA ligases, respectively, an observation potentially of use in selective adapter ligation for next-generation sequencing DNA library preparation. Differences in DNA binding affinity also make some ligases of greater utility in many applications. For example, hLig3 and PBCV1 DNA ligase fusions ligate many ends with high efficiency, but their tight nonspecific binding to sealed DNA, as evidenced by the aggressive proteinase K/SDS treatment needed to eliminate the gel shift when visualizing the ligation products, make them of less practical use in many instances than the weaker binding T4 DNA ligase. Finally, this study reveals intriguing results in end-joining reaction outcome from ligase to ligase, which are suggestive of structural features that result in different preferred end-joining substrates and mechanistic differences that control the likelihood of successful versus abortive ligation. Future work into the mechanistic underpinnings of end-joining ligation will explore these differences in greater depth.

## Supporting information

S1 Fig Protein gels to evaluate ligase purity. SDS-PAGE protein gels show purified wild type ligases, DNA-binding domain fusion ligases, and isolated binding domains used in this study. (A) Wild type ligases include: T3 DNA ligase, 39 kDa, lane 1; T4 DNA ligase, 55 kDa, lane 2; T7 DNA ligase, 41 kDa, lane 3; PBCV1 DNA ligase, 34 kDa, lane 4; hLig3, 98 kDa, lane 5; *E*. *coli* LigA, 74 kDa, lane 6. (B) DNA-binding domain fusion ligases include: PBCV1-Nterm-Sso7d, 44 kDa, lane 1; PBCV1-Cterm-Sso7d, 45 kDa, lane 2; PBCV1-Nterm-ZnF, 50 kDa, lane 3; PBCV1-Nterm-T4NTD, 51 kDa, lane 4. (C) Isolated binding domains include: Sso7d, 10 kDa, lane 1; ZnF, 16 kDa, lane 2; T4NTD, 16 kDa, lane 3. A protein standard ladder is included in the leftmost lane of each gel as a molecular weight marker (kDa).
(TIF)

S2 Fig Incubation of ligases with ssDNA to test for contaminating nuclease activity. Representative capillary electrophoresis traces of extended timepoint reaction to test for contaminating nuclease activity. A single stranded oligo with internal FAM label (5’-p-CTTCTAGGTTCCTATGA/FAM-T/TCTGGGACTGACCGAGCCTGACTCACAATTGATAGTTGCGTT-3’) was reacted with ligases used in this study. Reactions included 1 uM of the ligase and 100 nM of the substrate and T4 DNA ligase reaction buffer (50 mM Tris-HCl pH 7.5 @ 25°C, 1 mM ATP and 10 mM MgCl_2_) or NEBuffer 2 (10 mM Tris pH 7.9 @ 25°C, 50 mM NaCl, 10 mM MgCl_2_, 1 mM DTT). A 1-hour reaction time was used to provide an extended timepoint beyond the reaction time used for ligation experiments. No nuclease activity was observed for nearly all ligases in this study, with the exception of a trace amount of degradation (<5%) observed for hLig3.
(TIF)

S3 Fig Reaction endpoint and dsDNA nuclease contamination assessment for wild type ligases. Bar graphs depict the fraction of either ligated DNA (product) or abortive adenylylation (App) produced in an 18-hour sealing reaction with the indicated DNA substrate. Reactions included 1 μM of the DNA ligase, 100 nM of the substrate and reaction conditions consisting of NEBNext® Quick Ligation reaction buffer (66 mM Tris pH 7.6 @ 25°C, 10 mM MgCl_2_, 1 mM DTT, 1 mM ATP, 6% Polyethylene glycol (PEG 6000)). Ligation assays were performed with T3 DNA ligase, T4 DNA ligase, T7 DNA ligase, PBCV1 DNA ligase, hLig3, and *E*. *coli* DNA ligase A. Experiments were performed in triplicate; the plotted value is the average and the error bars represent the standard deviation across a minimum of 3 replicates.
(TIF)

S4 Fig Reaction endpoint and dsDNA nuclease contamination assessment for fusion ligases. Bar graphs depict the fraction of either ligated DNA (product) or abortive adenylylation (App) produced in an 18-hour sealing reaction with the indicated DNA substrate. Reactions included 1 μM of the DNA ligase, 100 nM of the substrate and reaction conditions consisting of NEBNext® Quick Ligation reaction buffer (66 mM Tris pH 7.6 @ 25°C, 10 mM MgCl_2_, 1 mM DTT, 1 mM ATP, 6% Polyethylene glycol (PEG 6000)). Ligation assays were performed with PBCV1-Nterm-Sso7d, PBCV1-Cterm-Sso7d, PBCV1-Nterm-ZnF, PBCV1-Nterm-T4NTD. Experiments were performed in triplicate; the plotted value is the average and the error bars represent the standard deviation across a minimum of 3 replicates.
(TIF)

S5 Fig Effect of monovalent cations on active blunt/cohesive-end ligases. Plotted data depicting the fraction of either sealed DNA (product) or abortive adenylylation (App) produced in a 20-minute sealing reaction with the indicated blunt/cohesive DNA substrate. Reactions included 1 μM of the DNA ligase, 100 nM of the substrate and reaction conditions consisting of either T4 DNA ligase reaction buffer + 150 mM NaCl (50 mM Tris-HCl pH 7.5 @ 25°C, 150 mM NaCl, 1 mM ATP and 10 mM MgCl_2_) or NEBNext^®^ Quick Ligation reaction buffer (66 mM Tris pH 7.6 @ 25°C, 10 mM MgCl_2_, 1 mM DTT, 150 mM NaCl, 1 mM ATP, 6% Polyethylene glycol (PEG 6000)). A) Blunt/cohesive substrate panel sealing performed by T4 DNA ligase. B) Blunt/cohesive substrate panel sealing performed by T3 DNA ligase. C) Blunt/cohesive substrate panel sealing performed by PBCV1 DNA ligase D) Blunt/cohesive substrate panel sealing performed by hLig3.
(TIF)

S6 Fig Separate addition of DNA binding domains to PBCV1 DNA ligase. Plotted data depicting the fraction of either sealed DNA (product) or abortive adenylylation (App) produced in a 20-minute sealing reaction with the indicated blunt/cohesive DNA substrate. Reactions included 1 μM of the DNA ligase, 100 nM of the substrate and reaction conditions consisting of either T4 DNA ligase reaction buffer (50 mM Tris-HCl pH 7.5 @ 25°C, 1 mM ATP and 10 mM MgCl_2_) or NEBNext^®^ Quick Ligation reaction buffer (66 mM Tris pH 7.6 @ 25°C, 10 mM MgCl_2_, 1 mM DTT, 1 mM ATP, 6% Polyethylene glycol (PEG 6000)).
(TIF)

S7 Fig Fluorescence anisotropy DNA binding curves. Fluorescence anisotropy DNA binding experiments. Using a 24mer dsDNA oligonucleotide substrate with a 3′-FAM label, the binding of various ligases and DNA binding domains (Sso7d, hLig3 ZnF) were determined through plotting the change in observed fluorescence anisotropy with a single binding model or hill binding equation (Eq 3, for Sso7d alone).
(TIF)

S1 Table Capillary gel electrophoresis panel raw data. All reactions were run as described in the main text, with the exception of the NAD^+^ dependent *E*. *Coli* ligase. In this case the buffer used was E. Coli DNA Ligase reaction buffer from NEB: 30 mM Tris-HCl, pH 8 @ 25°C,4 mM MgCl_2_, 26 μM NAD, 1 mM DTT, 50 μg/ml BSA. The equivalent Quick buffer was identical but also included 6% PEG. Buffers indicated as “+ NaCl” include 150 mM NaCl added to the base buffer indicated. QL = Quick Ligation. STDEV = standard deviation between replicates.
(DOCX)

## Abbreviations

AppDNA: 5′-adenylylated DNA
*E*. *coli* LigA: *Escherichia coli* DNA Ligase
PBCV1: *Paramecium bursaria* chlorella virus
hLig3: human DNA ligase III
hLig4: human DNA ligase IV
PEG: polyethylene glycol
NTase: nucleotidyl transferase domain
OBD: oligonucleotide binding domain
DBD: DNA binding domain
ZnF: Zinc Finger Domain
Sso7d: *Sulfolobus solfataricus* small double-stranded DNA binding protein
T4NTD: T4 DNA ligase N-terminal domain
FAM: 6-Carboxyfluorescein
ROX: carboxy-X-rhodamine
SBO: single base overhang
2BO: 2-base overhang
4BO: 4-base overhang
