# Supplementary figures and images for: Comparative analysis of the end-joining activity of several DNA ligases
Source: PLoS One. 2017 Dec 28;12(12):e0190062. doi: 10.1371/journal.pone.0190062 (PMC5746248; doi:10.1371/journal.pone.0190062)

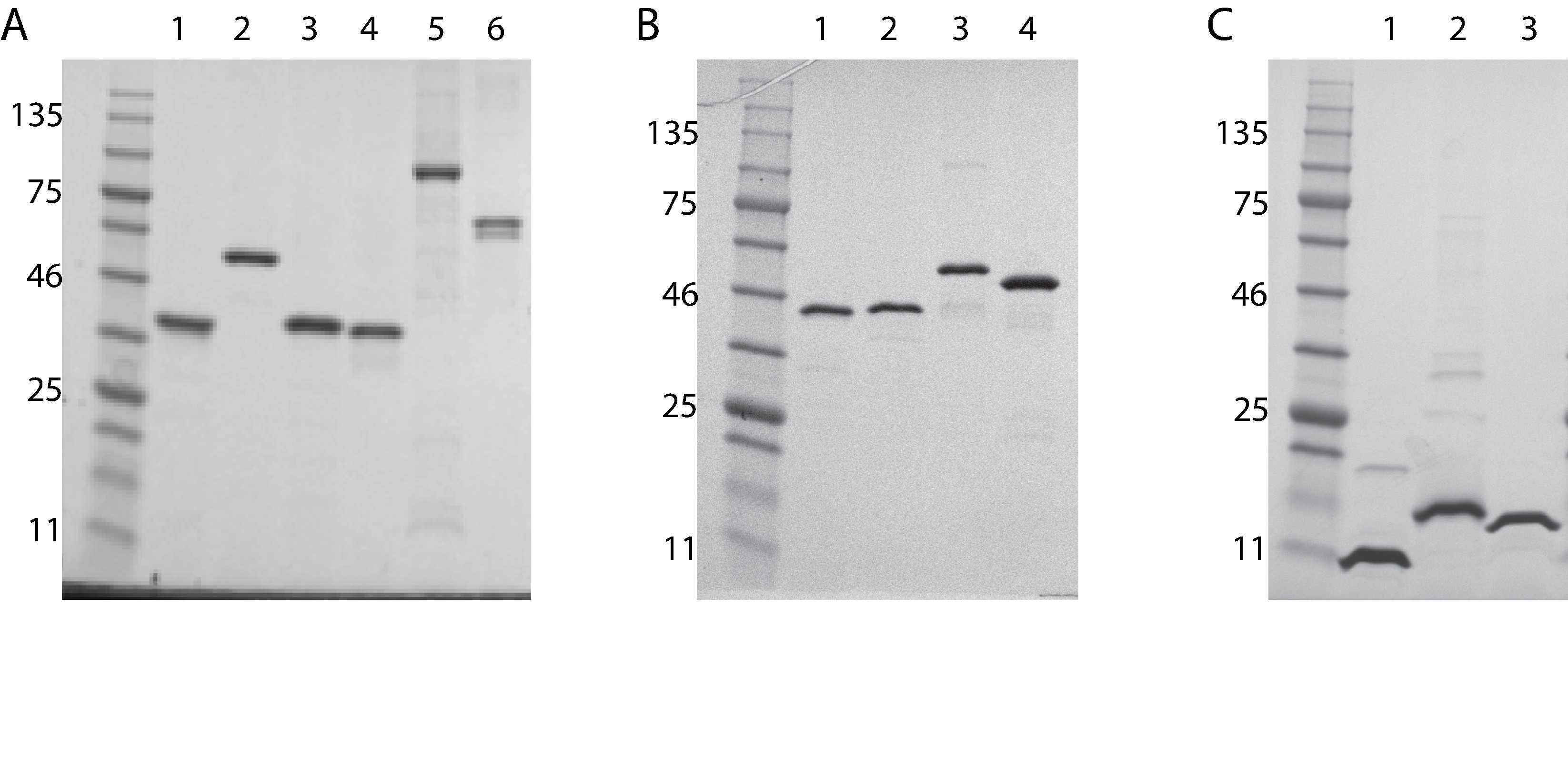

Supplement: S1 Fig — SDS-PAGE protein gels show purified wild type ligases, DNA-binding domain fusion ligases, and isolated binding domains used in this study. (A) Wild type ligases include: T3 DNA ligase, 39 kDa, lane 1; T4 DNA ligase, 55 kDa, lane 2; T7 DNA ligase, 41 kDa, lane 3; PBCV1 DNA ligase, 34 kDa, lane 4; hLig3, 98 kDa, lane 5; E. coli LigA, 74 kDa, lane 6. (B) DNA-binding domain fusion ligases include: PBCV1-Nterm-Sso7d, 44 kDa, lane 1; PBCV1-Cterm-Sso7d, 45 kDa, lane 2; PBCV1-Nterm-ZnF, 50 kDa, lane 3; PBCV1-Nterm-T4NTD, 51 kDa, lane 4. (C) Isolated binding domains include: Sso7d, 10 kDa, lane 1; ZnF, 16 kDa, lane 2; T4NTD, 16 kDa, lane 3. A protein standard ladder is included in the leftmost lane of each gel as a molecular weight marker (kDa). (TIF) [file pone.0190062.s001.tif]

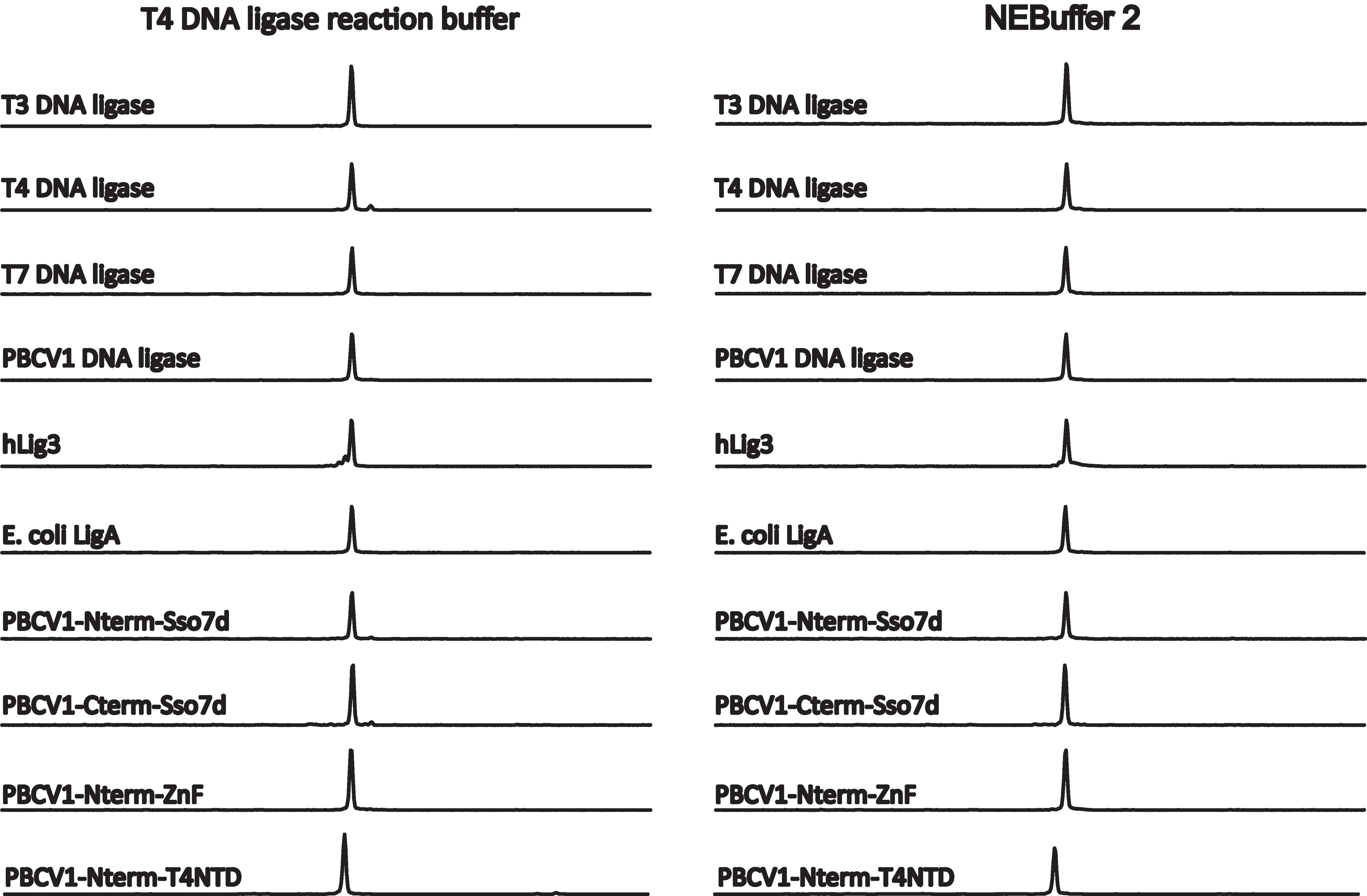

Supplement: S2 Fig — Representative capillary electrophoresis traces of extended timepoint reaction to test for contaminating nuclease activity. A single stranded oligo with internal FAM label (5’-p-CTTCTAGGTTCCTATGA/FAM-T/TCTGGGACTGACCGAGCCTGACTCACAATTGATAGTTGCGTT-3’) was reacted with ligases used in this study. Reactions included 1 uM of the ligase and 100 nM of the substrate and T4 DNA ligase reaction buffer (50 mM Tris-HCl pH 7.5 @ 25°C, 1 mM ATP and 10 mM MgCl2) or NEBuffer 2 (10 mM Tris pH 7.9 @ 25°C, 50 mM NaCl, 10 mM MgCl2, 1 mM DTT). A 1-hour reaction time was used to provide an extended timepoint beyond the reaction time used for ligation experiments. No nuclease activity was observed for nearly all ligases in this study, with the exception of a trace amount of degradation (<5%) observed for hLig3. (TIF) [file pone.0190062.s002.tif]

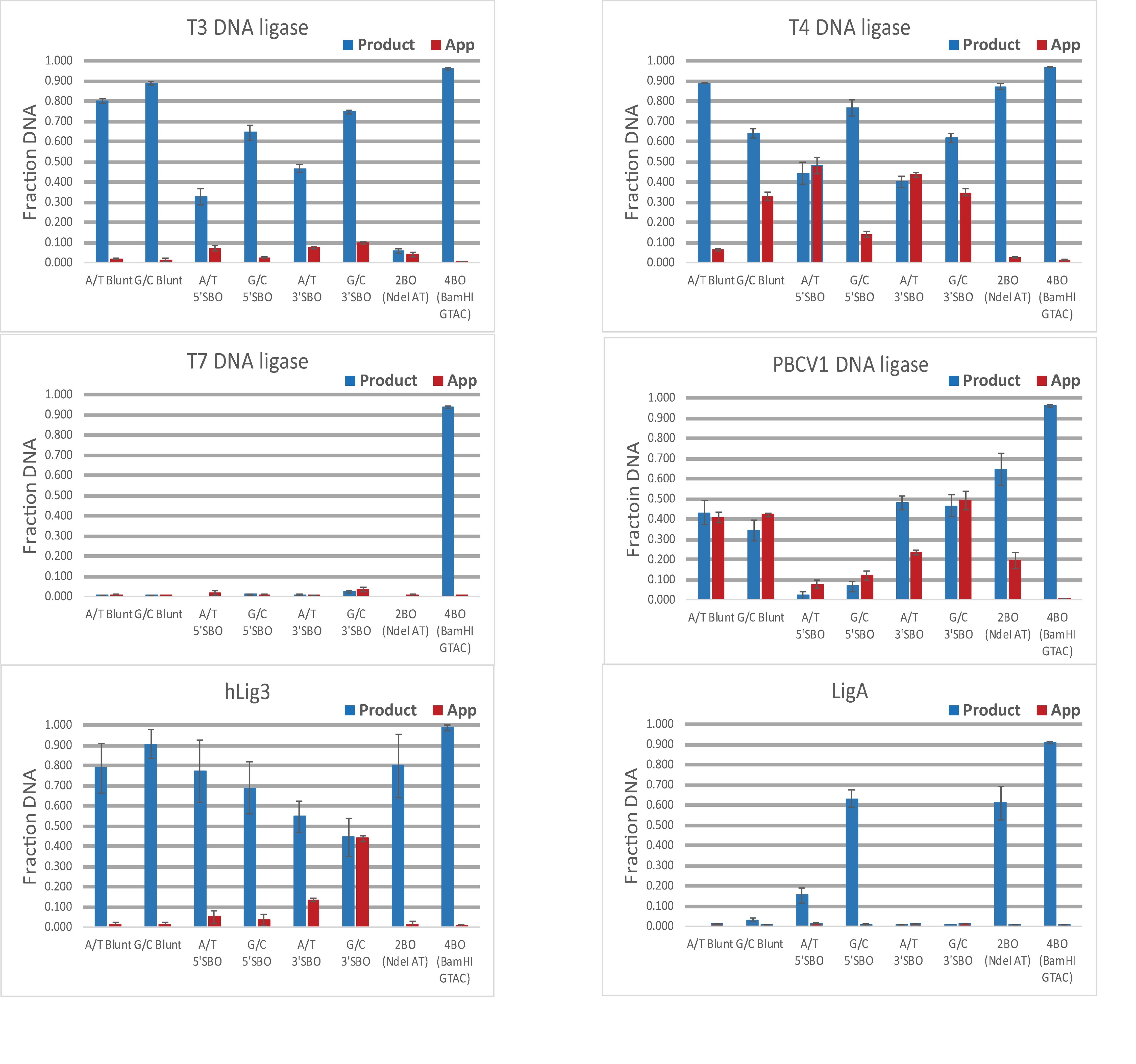

Supplement: S3 Fig — Bar graphs depict the fraction of either ligated DNA (product) or abortive adenylylation (App) produced in an 18-hour sealing reaction with the indicated DNA substrate. Reactions included 1 μM of the DNA ligase, 100 nM of the substrate and reaction conditions consisting of NEBNext® Quick Ligation reaction buffer (66 mM Tris pH 7.6 @ 25°C, 10 mM MgCl2, 1 mM DTT, 1 mM ATP, 6% Polyethylene glycol (PEG 6000)). Ligation assays were performed with T3 DNA ligase, T4 DNA ligase, T7 DNA ligase, PBCV1 DNA ligase, hLig3, and E. coli DNA ligase A. Experiments were performed in triplicate; the plotted value is the average and the error bars represent the standard deviation across a minimum of 3 replicates. (TIF) [file pone.0190062.s003.tif]

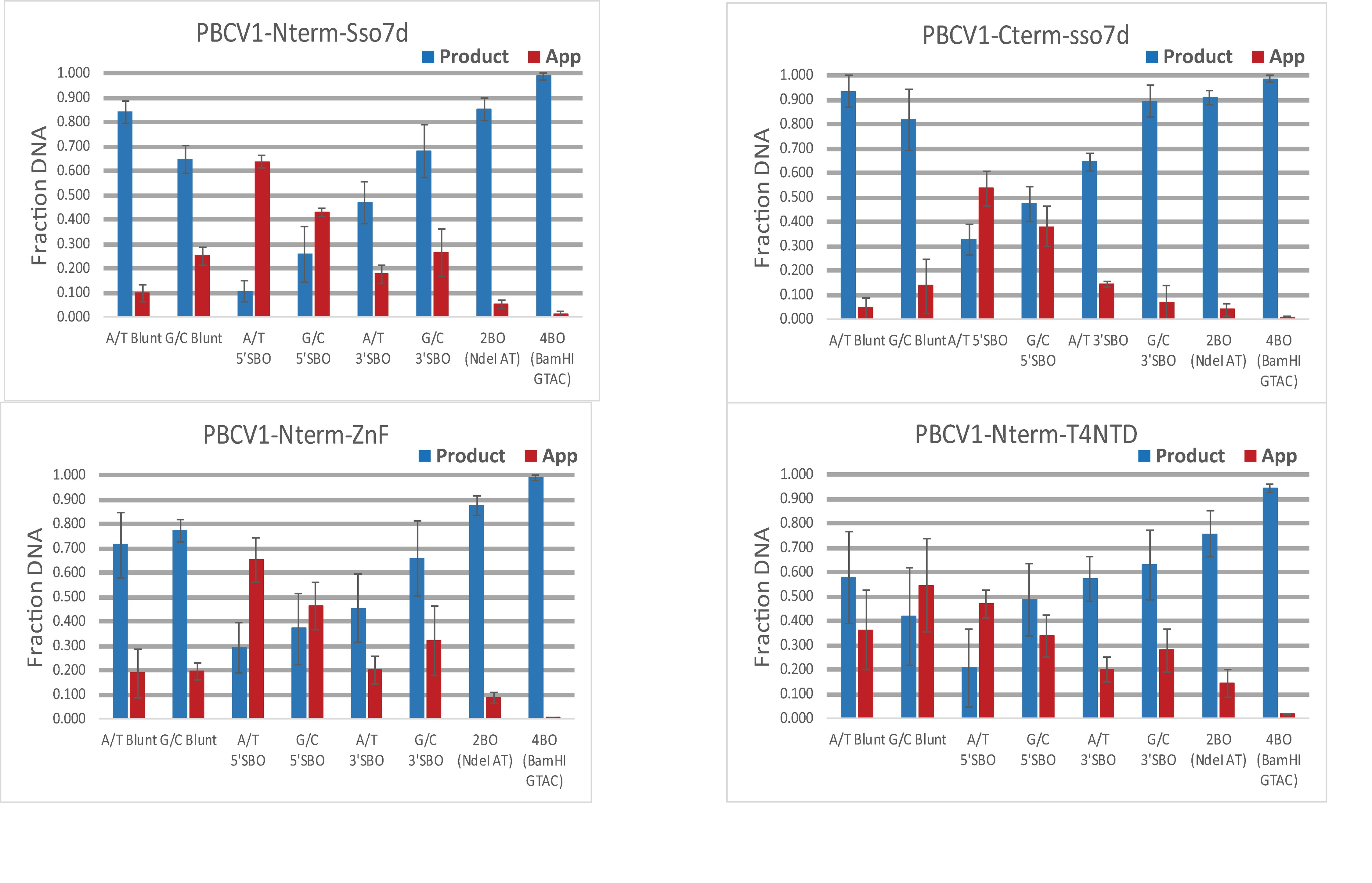

Supplement: S4 Fig — Bar graphs depict the fraction of either ligated DNA (product) or abortive adenylylation (App) produced in an 18-hour sealing reaction with the indicated DNA substrate. Reactions included 1 μM of the DNA ligase, 100 nM of the substrate and reaction conditions consisting of NEBNext® Quick Ligation reaction buffer (66 mM Tris pH 7.6 @ 25°C, 10 mM MgCl2, 1 mM DTT, 1 mM ATP, 6% Polyethylene glycol (PEG 6000)). Ligation assays were performed with PBCV1-Nterm-Sso7d, PBCV1-Cterm-Sso7d, PBCV1-Nterm-ZnF, PBCV1-Nterm-T4NTD. Experiments were performed in triplicate; the plotted value is the average and the error bars represent the standard deviation across a minimum of 3 replicates. (TIF) [file pone.0190062.s004.tif]

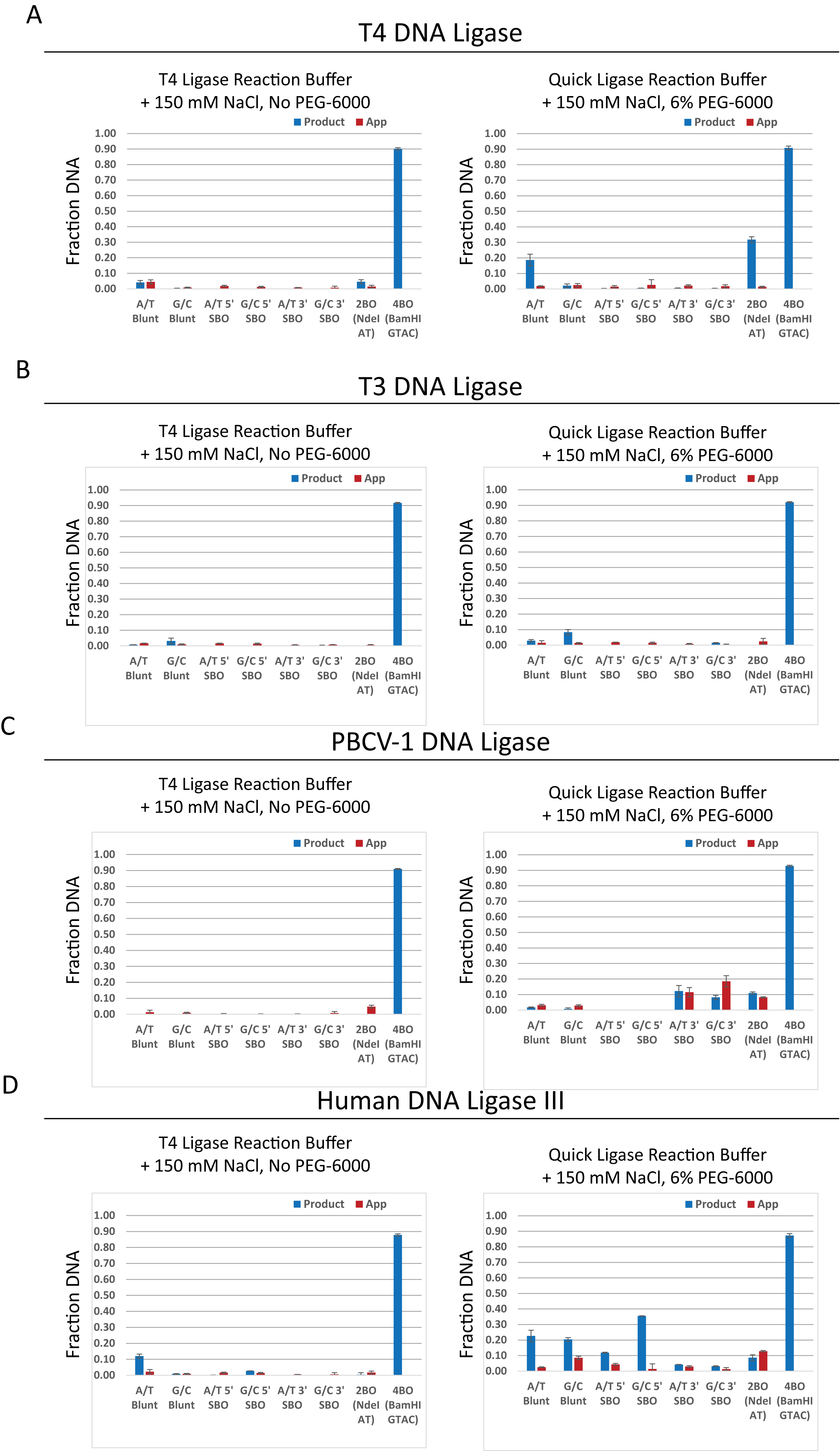

Supplement: S5 Fig — Plotted data depicting the fraction of either sealed DNA (product) or abortive adenylylation (App) produced in a 20-minute sealing reaction with the indicated blunt/cohesive DNA substrate. Reactions included 1 μM of the DNA ligase, 100 nM of the substrate and reaction conditions consisting of either T4 DNA ligase reaction buffer + 150 mM NaCl (50 mM Tris-HCl pH 7.5 @ 25°C, 150 mM NaCl, 1 mM ATP and 10 mM MgCl2) or NEBNext® Quick Ligation reaction buffer (66 mM Tris pH 7.6 @ 25°C, 10 mM MgCl2, 1 mM DTT, 150 mM NaCl, 1 mM ATP, 6% Polyethylene glycol (PEG 6000)). A) Blunt/cohesive substrate panel sealing performed by T4 DNA ligase. B) Blunt/cohesive substrate panel sealing performed by T3 DNA ligase. C) Blunt/cohesive substrate panel sealing performed by PBCV1 DNA ligase D) Blunt/cohesive substrate panel sealing performed by hLig3. (TIF) [file pone.0190062.s005.tif]

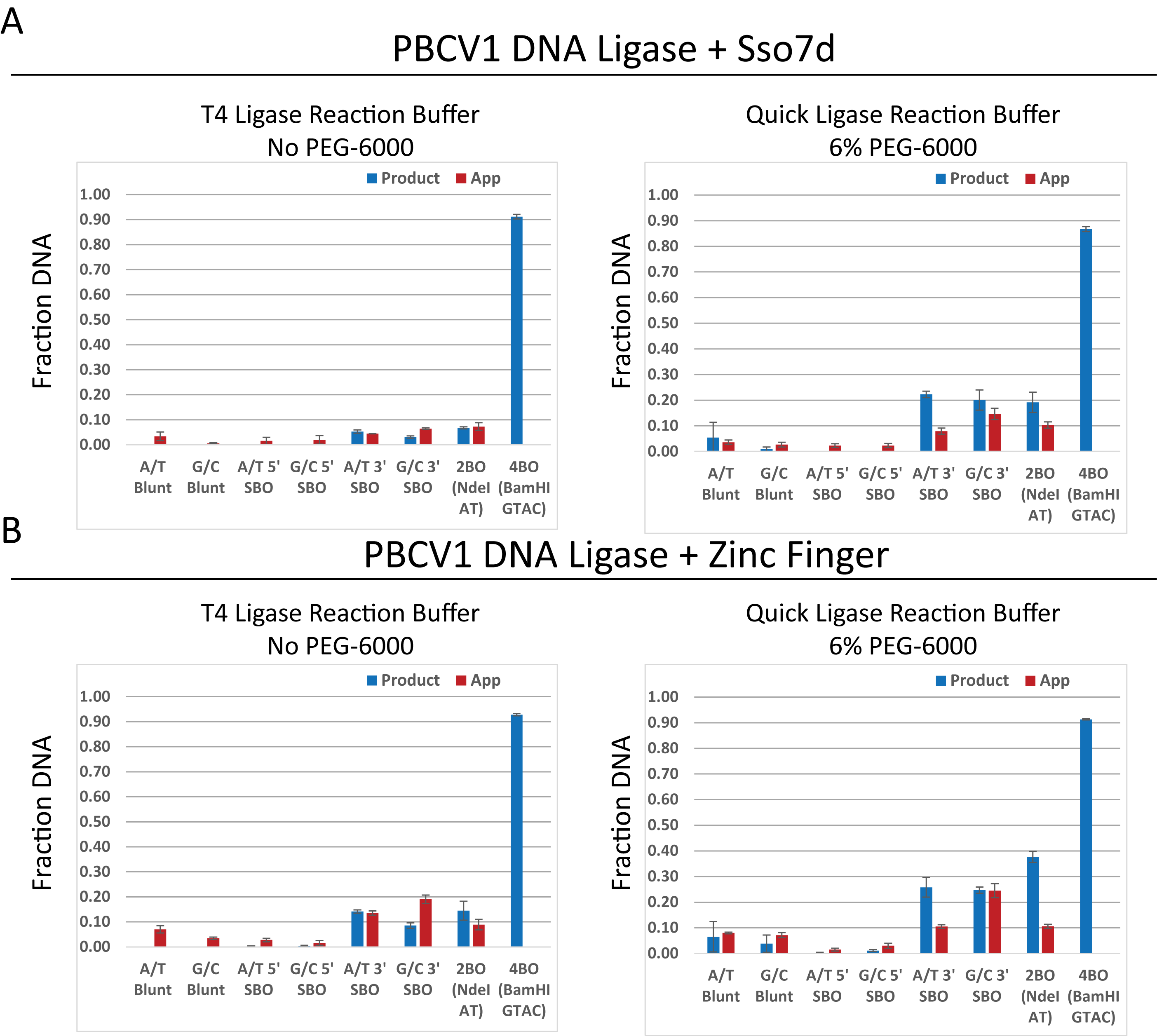

Supplement: S6 Fig — Plotted data depicting the fraction of either sealed DNA (product) or abortive adenylylation (App) produced in a 20-minute sealing reaction with the indicated blunt/cohesive DNA substrate. Reactions included 1 μM of the DNA ligase, 100 nM of the substrate and reaction conditions consisting of either T4 DNA ligase reaction buffer (50 mM Tris-HCl pH 7.5 @ 25°C, 1 mM ATP and 10 mM MgCl2) or NEBNext® Quick Ligation reaction buffer (66 mM Tris pH 7.6 @ 25°C, 10 mM MgCl2, 1 mM DTT, 1 mM ATP, 6% Polyethylene glycol (PEG 6000)). (TIF) [file pone.0190062.s006.tif]
