# Supplementary material for: Comparative analysis of the end-joining activity of several DNA ligases
Source: PLoS One. 2017 Dec 28;12(12):e0190062. doi: 10.1371/journal.pone.0190062 (PMC5746248; doi:10.1371/journal.pone.0190062)
Supplement: S1 Table — All reactions were run as described in the main text, with the exception of the NAD+ dependent E. Coli ligase. In this case the buffer used was E. Coli DNA Ligase reaction buffer from NEB: 30 mM Tris-HCl, pH 8 @ 25°C,4 mM MgCl2, 26 μM NAD, 1 mM DTT, 50 μg/ml BSA. The equivalent Quick buffer was identical but also included 6% PEG. Buffers indicated as “+ NaCl” include 150 mM NaCl added to the base buffer indicated. QL = Quick Ligation. STDEV = standard deviation between replicates. (DOCX) [file pone.0190062.s008.docx]

| **DNA Ligase** | **[Ligase]** | **Buffer** | **Substrate** | **Fraction Product** | **Product STDEV** | **Fraction AppDNA** | **AppDNA STDEV** |
| --- | --- | --- | --- | --- | --- | --- | --- |
| T4 | 100 nM | T4 Buffer | A/T Blunt | 0.272 | 0.023 | 0.021 | 0.021 |
| T4 | 100 nM | T4 Buffer | G/C Blunt | 0.037 | 0.007 | 0.022 | 0.004 |
| T4 | 100 nM | T4 Buffer | A/T 5' SBO | 0.000 | 0.000 | 0.032 | 0.009 |
| T4 | 100 nM | T4 Buffer | G/C 5' SBO | 0.015 | 0.002 | 0.024 | 0.005 |
| T4 | 100 nM | T4 Buffer | A/T 3' SBO | 0.000 | 0.000 | 0.075 | 0.004 |
| T4 | 100 nM | T4 Buffer | G/C 3' SBO | 0.023 | 0.011 | 0.060 | 0.066 |
| T4 | 100 nM | T4 Buffer | 2BO | 0.287 | 0.037 | 0.032 | 0.015 |
| T4 | 100 nM | T4 Buffer | 4BO | 0.920 | 0.001 | 0.000 | 0.000 |
| T4 | 1000 nM | T4 Buffer | A/T Blunt | 0.637 | 0.036 | 0.084 | 0.029 |
| T4 | 1000 nM | T4 Buffer | G/C Blunt | 0.205 | 0.010 | 0.242 | 0.013 |
| T4 | 1000 nM | T4 Buffer | A/T 5' SBO | 0.000 | 0.000 | 0.046 | 0.022 |
| T4 | 1000 nM | T4 Buffer | G/C 5' SBO | 0.081 | 0.020 | 0.155 | 0.046 |
| T4 | 1000 nM | T4 Buffer | A/T 3' SBO | 0.041 | 0.003 | 0.364 | 0.034 |
| T4 | 1000 nM | T4 Buffer | G/C 3' SBO | 0.069 | 0.006 | 0.112 | 0.017 |
| T4 | 1000 nM | T4 Buffer | 2BO | 0.731 | 0.056 | 0.016 | 0.014 |
| T4 | 1000 nM | T4 Buffer | 4BO | 0.909 | 0.050 | 0.000 | 0.000 |
| T4 | 100 nM | QL Buffer | A/T Blunt | 0.649 | 0.016 | 0.053 | 0.011 |
| T4 | 100 nM | QL Buffer | G/C Blunt | 0.296 | 0.029 | 0.122 | 0.001 |
| T4 | 100 nM | QL Buffer | A/T 5' SBO | 0.002 | 0.004 | 0.059 | 0.050 |
| T4 | 100 nM | QL Buffer | G/C 5' SBO | 0.170 | 0.022 | 0.141 | 0.015 |
| T4 | 100 nM | QL Buffer | A/T 3' SBO | 0.104 | 0.004 | 0.284 | 0.010 |
| T4 | 100 nM | QL Buffer | G/C 3' SBO | 0.160 | 0.009 | 0.099 | 0.007 |
| T4 | 100 nM | QL Buffer | 2BO | 0.675 | 0.001 | 0.032 | 0.010 |
| T4 | 100 nM | QL Buffer | 4BO | 0.926 | 0.006 | 0.000 | 0.000 |
| T4 | 1000 nM | QL Buffer | A/T Blunt | 0.799 | 0.015 | 0.043 | 0.009 |
| T4 | 1000 nM | QL Buffer | G/C Blunt | 0.531 | 0.023 | 0.241 | 0.011 |
| T4 | 1000 nM | QL Buffer | A/T 5' SBO | 0.135 | 0.115 | 0.127 | 0.034 |
| T4 | 1000 nM | QL Buffer | G/C 5' SBO | 0.225 | 0.010 | 0.595 | 0.010 |
| T4 | 1000 nM | QL Buffer | A/T 3' SBO | 0.421 | 0.011 | 0.272 | 0.017 |
| T4 | 1000 nM | QL Buffer | G/C 3' SBO | 0.207 | 0.107 | 0.138 | 0.028 |
| T4 | 1000 nM | QL Buffer | 2BO | 0.891 | 0.008 | 0.014 | 0.012 |
| T4 | 1000 nM | QL Buffer | 4BO | 0.942 | 0.001 | 0.000 | 0.000 |
| PBCV1 | 100 nM | T4 Buffer | A/T Blunt | 0.001 | 0.002 | 0.018 | 0.005 |
| PBCV1 | 100 nM | T4 Buffer | G/C Blunt | 0.000 | 0.000 | 0.002 | 0.004 |
| PBCV1 | 100 nM | T4 Buffer | A/T 5' SBO | 0.000 | 0.000 | 0.022 | 0.006 |
| PBCV1 | 100 nM | T4 Buffer | G/C 5' SBO | 0.000 | 0.000 | 0.035 | 0.002 |
| PBCV1 | 100 nM | T4 Buffer | A/T 3' SBO | 0.021 | 0.003 | 0.048 | 0.005 |
| PBCV1 | 100 nM | T4 Buffer | G/C 3' SBO | 0.012 | 0.004 | 0.059 | 0.024 |
| PBCV1 | 100 nM | T4 Buffer | 2BO | 0.019 | 0.008 | 0.022 | 0.003 |
| PBCV1 | 100 nM | T4 Buffer | 4BO | 0.911 | 0.004 | 0.000 | 0.000 |
| PBCV1 | 1000 nM | T4 Buffer | A/T Blunt | 0.031 | 0.005 | 0.083 | 0.016 |
| PBCV1 | 1000 nM | T4 Buffer | G/C Blunt | 0.014 | 0.007 | 0.051 | 0.017 |
| PBCV1 | 1000 nM | T4 Buffer | A/T 5' SBO | 0.000 | 0.000 | 0.020 | 0.019 |
| PBCV1 | 1000 nM | T4 Buffer | G/C 5' SBO | 0.000 | 0.000 | 0.009 | 0.003 |
| PBCV1 | 1000 nM | T4 Buffer | A/T 3' SBO | 0.139 | 0.006 | 0.214 | 0.003 |
| PBCV1 | 1000 nM | T4 Buffer | G/C 3' SBO | 0.091 | 0.012 | 0.351 | 0.008 |
| PBCV1 | 1000 nM | T4 Buffer | 2BO | 0.169 | 0.008 | 0.150 | 0.012 |
| PBCV1 | 1000 nM | T4 Buffer | 4BO | 0.927 | 0.004 | 0.000 | 0.000 |
| PBCV1 | 100 nM | QL Buffer | A/T Blunt | 0.015 | 0.001 | 0.033 | 0.011 |
| PBCV1 | 100 nM | QL Buffer | G/C Blunt | 0.007 | 0.002 | 0.020 | 0.006 |
| PBCV1 | 100 nM | QL Buffer | A/T 5' SBO | 0.000 | 0.000 | 0.026 | 0.014 |
| PBCV1 | 100 nM | QL Buffer | G/C 5' SBO | 0.000 | 0.000 | 0.014 | 0.005 |
| PBCV1 | 100 nM | QL Buffer | A/T 3' SBO | 0.095 | 0.015 | 0.070 | 0.002 |
| PBCV1 | 100 nM | QL Buffer | G/C 3' SBO | 0.054 | 0.014 | 0.107 | 0.015 |
| PBCV1 | 100 nM | QL Buffer | 2BO | 0.063 | 0.013 | 0.032 | 0.004 |
| PBCV1 | 100 nM | QL Buffer | 4BO | 0.918 | 0.000 | 0.000 | 0.000 |
| PBCV1 | 1000 nM | QL Buffer | A/T Blunt | 0.077 | 0.014 | 0.119 | 0.014 |
| PBCV1 | 1000 nM | QL Buffer | G/C Blunt | 0.033 | 0.007 | 0.088 | 0.011 |
| PBCV1 | 1000 nM | QL Buffer | A/T 5' SBO | 0.000 | 0.000 | 0.031 | 0.011 |
| PBCV1 | 1000 nM | QL Buffer | G/C 5' SBO | 0.000 | 0.000 | 0.014 | 0.013 |
| PBCV1 | 1000 nM | QL Buffer | A/T 3' SBO | 0.296 | 0.014 | 0.242 | 0.008 |
| PBCV1 | 1000 nM | QL Buffer | G/C 3' SBO | 0.208 | 0.003 | 0.398 | 0.007 |
| PBCV1 | 1000 nM | QL Buffer | 2BO | 0.339 | 0.010 | 0.170 | 0.015 |
| PBCV1 | 1000 nM | QL Buffer | 4BO | 0.925 | 0.002 | 0.000 | 0.000 |
| PBCV1 N-Term Sso7d | 100 nM | T4 Buffer | A/T Blunt | 0.000 | 0.000 | 0.020 | 0.005 |
| PBCV1 N-Term Sso7d | 100 nM | T4 Buffer | G/C Blunt | 0.000 | 0.000 | 0.019 | 0.011 |
| PBCV1 N-Term Sso7d | 100 nM | T4 Buffer | A/T 5' SBO | 0.000 | 0.000 | 0.018 | 0.003 |
| PBCV1 N-Term Sso7d | 100 nM | T4 Buffer | G/C 5' SBO | 0.000 | 0.000 | 0.020 | 0.004 |
| PBCV1 N-Term Sso7d | 100 nM | T4 Buffer | A/T 3' SBO | 0.005 | 0.001 | 0.034 | 0.009 |
| PBCV1 N-Term Sso7d | 100 nM | T4 Buffer | G/C 3' SBO | 0.000 | 0.000 | 0.037 | 0.004 |
| PBCV1 N-Term Sso7d | 100 nM | T4 Buffer | 2BO | 0.000 | 0.000 | 0.029 | 0.010 |
| PBCV1 N-Term Sso7d | 100 nM | T4 Buffer | 4BO | 0.918 | 0.005 | 0.000 | 0.000 |
| PBCV1 N-Term Sso7d | 1000 nM | T4 Buffer | A/T Blunt | 0.030 | 0.005 | 0.150 | 0.015 |
| PBCV1 N-Term Sso7d | 1000 nM | T4 Buffer | G/C Blunt | 0.007 | 0.007 | 0.113 | 0.006 |
| PBCV1 N-Term Sso7d | 1000 nM | T4 Buffer | A/T 5' SBO | 0.000 | 0.000 | 0.023 | 0.011 |
| PBCV1 N-Term Sso7d | 1000 nM | T4 Buffer | G/C 5' SBO | 0.000 | 0.000 | 0.013 | 0.003 |
| PBCV1 N-Term Sso7d | 1000 nM | T4 Buffer | A/T 3' SBO | 0.061 | 0.007 | 0.193 | 0.010 |
| PBCV1 N-Term Sso7d | 1000 nM | T4 Buffer | G/C 3' SBO | 0.033 | 0.004 | 0.270 | 0.013 |
| PBCV1 N-Term Sso7d | 1000 nM | T4 Buffer | 2BO | 0.037 | 0.003 | 0.249 | 0.011 |
| PBCV1 N-Term Sso7d | 1000 nM | T4 Buffer | 4BO | 0.927 | 0.003 | 0.000 | 0.000 |
| PBCV1 N-Term Sso7d | 100 nM | QL Buffer | A/T Blunt | 0.021 | 0.005 | 0.039 | 0.007 |
| PBCV1 N-Term Sso7d | 100 nM | QL Buffer | G/C Blunt | 0.006 | 0.006 | 0.013 | 0.004 |
| PBCV1 N-Term Sso7d | 100 nM | QL Buffer | A/T 5' SBO | 0.000 | 0.000 | 0.011 | 0.010 |
| PBCV1 N-Term Sso7d | 100 nM | QL Buffer | G/C 5' SBO | 0.000 | 0.000 | 0.013 | 0.002 |
| PBCV1 N-Term Sso7d | 100 nM | QL Buffer | A/T 3' SBO | 0.062 | 0.046 | 0.078 | 0.025 |
| PBCV1 N-Term Sso7d | 100 nM | QL Buffer | G/C 3' SBO | 0.026 | 0.007 | 0.068 | 0.054 |
| PBCV1 N-Term Sso7d | 100 nM | QL Buffer | 2BO | 0.024 | 0.004 | 0.063 | 0.007 |
| PBCV1 N-Term Sso7d | 100 nM | QL Buffer | 4BO | 0.905 | 0.003 | 0.000 | 0.000 |
| PBCV1 N-Term Sso7d | 1000 nM | QL Buffer | A/T Blunt | 0.424 | 0.036 | 0.292 | 0.022 |
| PBCV1 N-Term Sso7d | 1000 nM | QL Buffer | G/C Blunt | 0.352 | 0.022 | 0.456 | 0.022 |
| PBCV1 N-Term Sso7d | 1000 nM | QL Buffer | A/T 5' SBO | 0.033 | 0.004 | 0.222 | 0.032 |
| PBCV1 N-Term Sso7d | 1000 nM | QL Buffer | G/C 5' SBO | 0.095 | 0.006 | 0.306 | 0.049 |
| PBCV1 N-Term Sso7d | 1000 nM | QL Buffer | A/T 3' SBO | 0.524 | 0.073 | 0.162 | 0.129 |
| PBCV1 N-Term Sso7d | 1000 nM | QL Buffer | G/C 3' SBO | 0.408 | 0.022 | 0.449 | 0.030 |
| PBCV1 N-Term Sso7d | 1000 nM | QL Buffer | 2BO | 0.692 | 0.046 | 0.257 | 0.028 |
| PBCV1 N-Term Sso7d | 1000 nM | QL Buffer | 4BO | 0.923 | 0.008 | 0.000 | 0.000 |
| PBCV1 C-Term Sso7d | 100 nM | T4 Buffer | A/T Blunt | 0.000 | 0.000 | 0.016 | 0.008 |
| PBCV1 C-Term Sso7d | 100 nM | T4 Buffer | G/C Blunt | 0.000 | 0.000 | 0.013 | 0.011 |
| PBCV1 C-Term Sso7d | 100 nM | T4 Buffer | A/T 5' SBO | 0.000 | 0.000 | 0.020 | 0.004 |
| PBCV1 C-Term Sso7d | 100 nM | T4 Buffer | G/C 5' SBO | 0.000 | 0.000 | 0.037 | 0.013 |
| PBCV1 C-Term Sso7d | 100 nM | T4 Buffer | A/T 3' SBO | 0.002 | 0.003 | 0.051 | 0.021 |
| PBCV1 C-Term Sso7d | 100 nM | T4 Buffer | G/C 3' SBO | 0.000 | 0.000 | 0.044 | 0.005 |
| PBCV1 C-Term Sso7d | 100 nM | T4 Buffer | 2BO | 0.004 | 0.004 | 0.043 | 0.015 |
| PBCV1 C-Term Sso7d | 100 nM | T4 Buffer | 4BO | 0.902 | 0.003 | 0.000 | 0.000 |
| PBCV1 C-Term Sso7d | 1000 nM | T4 Buffer | A/T Blunt | 0.020 | 0.004 | 0.165 | 0.007 |
| PBCV1 C-Term Sso7d | 1000 nM | T4 Buffer | G/C Blunt | 0.000 | 0.000 | 0.116 | 0.006 |
| PBCV1 C-Term Sso7d | 1000 nM | T4 Buffer | A/T 5' SBO | 0.000 | 0.000 | 0.031 | 0.023 |
| PBCV1 C-Term Sso7d | 1000 nM | T4 Buffer | G/C 5' SBO | 0.000 | 0.000 | 0.013 | 0.014 |
| PBCV1 C-Term Sso7d | 1000 nM | T4 Buffer | A/T 3' SBO | 0.049 | 0.006 | 0.197 | 0.033 |
| PBCV1 C-Term Sso7d | 1000 nM | T4 Buffer | G/C 3' SBO | 0.029 | 0.004 | 0.322 | 0.089 |
| PBCV1 C-Term Sso7d | 1000 nM | T4 Buffer | 2BO | 0.074 | 0.009 | 0.400 | 0.025 |
| PBCV1 C-Term Sso7d | 1000 nM | T4 Buffer | 4BO | 0.928 | 0.006 | 0.000 | 0.000 |
| PBCV1 C-Term Sso7d | 100 nM | QL Buffer | A/T Blunt | 0.006 | 0.010 | 0.035 | 0.014 |
| PBCV1 C-Term Sso7d | 100 nM | QL Buffer | G/C Blunt | 0.000 | 0.000 | 0.029 | 0.014 |
| PBCV1 C-Term Sso7d | 100 nM | QL Buffer | A/T 5' SBO | 0.000 | 0.000 | 0.013 | 0.012 |
| PBCV1 C-Term Sso7d | 100 nM | QL Buffer | G/C 5' SBO | 0.000 | 0.000 | 0.022 | 0.025 |
| PBCV1 C-Term Sso7d | 100 nM | QL Buffer | A/T 3' SBO | 0.036 | 0.002 | 0.075 | 0.015 |
| PBCV1 C-Term Sso7d | 100 nM | QL Buffer | G/C 3' SBO | 0.070 | 0.085 | 0.342 | 0.424 |
| PBCV1 C-Term Sso7d | 100 nM | QL Buffer | 2BO | 0.025 | 0.001 | 0.067 | 0.004 |
| PBCV1 C-Term Sso7d | 100 nM | QL Buffer | 4BO | 0.895 | 0.003 | 0.000 | 0.000 |
| PBCV1 C-Term Sso7d | 1000 nM | QL Buffer | A/T Blunt | 0.345 | 0.061 | 0.467 | 0.024 |
| PBCV1 C-Term Sso7d | 1000 nM | QL Buffer | G/C Blunt | 0.212 | 0.012 | 0.618 | 0.002 |
| PBCV1 C-Term Sso7d | 1000 nM | QL Buffer | A/T 5' SBO | 0.000 | 0.000 | 0.097 | 0.018 |
| PBCV1 C-Term Sso7d | 1000 nM | QL Buffer | G/C 5' SBO | 0.015 | 0.002 | 0.153 | 0.014 |
| PBCV1 C-Term Sso7d | 1000 nM | QL Buffer | A/T 3' SBO | 0.358 | 0.027 | 0.319 | 0.014 |
| PBCV1 C-Term Sso7d | 1000 nM | QL Buffer | G/C 3' SBO | 0.291 | 0.015 | 0.564 | 0.023 |
| PBCV1 C-Term Sso7d | 1000 nM | QL Buffer | 2BO | 0.524 | 0.021 | 0.424 | 0.030 |
| PBCV1 C-Term Sso7d | 1000 nM | QL Buffer | 4BO | 0.929 | 0.004 | 0.000 | 0.000 |
| PBCV1 N-Term ZnF | 100 nM | T4 Buffer | A/T Blunt | 0.010 | 0.004 | 0.033 | 0.011 |
| PBCV1 N-Term ZnF | 100 nM | T4 Buffer | G/C Blunt | 0.015 | 0.007 | 0.073 | 0.025 |
| PBCV1 N-Term ZnF | 100 nM | T4 Buffer | A/T 5' SBO | 0.000 | 0.000 | 0.022 | 0.004 |
| PBCV1 N-Term ZnF | 100 nM | T4 Buffer | G/C 5' SBO | 0.000 | 0.000 | 0.025 | 0.024 |
| PBCV1 N-Term ZnF | 100 nM | T4 Buffer | A/T 3' SBO | 0.021 | 0.003 | 0.065 | 0.013 |
| PBCV1 N-Term ZnF | 100 nM | T4 Buffer | G/C 3' SBO | 0.017 | 0.004 | 0.103 | 0.026 |
| PBCV1 N-Term ZnF | 100 nM | T4 Buffer | 2BO | 0.022 | 0.007 | 0.063 | 0.015 |
| PBCV1 N-Term ZnF | 100 nM | T4 Buffer | 4BO | 0.863 | 0.036 | 0.000 | 0.000 |
| PBCV1 N-Term ZnF | 1000 nM | T4 Buffer | A/T Blunt | 0.158 | 0.016 | 0.365 | 0.028 |
| PBCV1 N-Term ZnF | 1000 nM | T4 Buffer | G/C Blunt | 0.136 | 0.018 | 0.624 | 0.034 |
| PBCV1 N-Term ZnF | 1000 nM | T4 Buffer | A/T 5' SBO | 0.020 | 0.005 | 0.379 | 0.034 |
| PBCV1 N-Term ZnF | 1000 nM | T4 Buffer | G/C 5' SBO | 0.026 | 0.004 | 0.340 | 0.013 |
| PBCV1 N-Term ZnF | 1000 nM | T4 Buffer | A/T 3' SBO | 0.196 | 0.017 | 0.363 | 0.021 |
| PBCV1 N-Term ZnF | 1000 nM | T4 Buffer | G/C 3' SBO | 0.140 | 0.007 | 0.597 | 0.024 |
| PBCV1 N-Term ZnF | 1000 nM | T4 Buffer | 2BO | 0.238 | 0.027 | 0.441 | 0.021 |
| PBCV1 N-Term ZnF | 1000 nM | T4 Buffer | 4BO | 0.866 | 0.116 | 0.000 | 0.000 |
| PBCV1 N-Term ZnF | 100 nM | QL Buffer | A/T Blunt | 0.078 | 0.011 | 0.043 | 0.003 |
| PBCV1 N-Term ZnF | 100 nM | QL Buffer | G/C Blunt | 0.072 | 0.011 | 0.078 | 0.006 |
| PBCV1 N-Term ZnF | 100 nM | QL Buffer | A/T 5' SBO | 0.007 | 0.002 | 0.047 | 0.012 |
| PBCV1 N-Term ZnF | 100 nM | QL Buffer | G/C 5' SBO | 0.020 | 0.002 | 0.036 | 0.003 |
| PBCV1 N-Term ZnF | 100 nM | QL Buffer | A/T 3' SBO | 0.122 | 0.014 | 0.062 | 0.010 |
| PBCV1 N-Term ZnF | 100 nM | QL Buffer | G/C 3' SBO | 0.110 | 0.006 | 0.115 | 0.005 |
| PBCV1 N-Term ZnF | 100 nM | QL Buffer | 2BO | 0.113 | 0.007 | 0.047 | 0.004 |
| PBCV1 N-Term ZnF | 100 nM | QL Buffer | 4BO | 0.897 | 0.006 | 0.000 | 0.000 |
| PBCV1 N-Term ZnF | 1000 nM | QL Buffer | A/T Blunt | 0.290 | 0.031 | 0.367 | 0.010 |
| PBCV1 N-Term ZnF | 1000 nM | QL Buffer | G/C Blunt | 0.268 | 0.011 | 0.555 | 0.005 |
| PBCV1 N-Term ZnF | 1000 nM | QL Buffer | A/T 5' SBO | 0.035 | 0.007 | 0.663 | 0.022 |
| PBCV1 N-Term ZnF | 1000 nM | QL Buffer | G/C 5' SBO | 0.051 | 0.006 | 0.579 | 0.057 |
| PBCV1 N-Term ZnF | 1000 nM | QL Buffer | A/T 3' SBO | 0.473 | 0.012 | 0.230 | 0.015 |
| PBCV1 N-Term ZnF | 1000 nM | QL Buffer | G/C 3' SBO | 0.362 | 0.010 | 0.512 | 0.014 |
| PBCV1 N-Term ZnF | 1000 nM | QL Buffer | 2BO | 0.641 | 0.069 | 0.318 | 0.060 |
| PBCV1 N-Term ZnF | 1000 nM | QL Buffer | 4BO | 0.955 | 0.003 | 0.000 | 0.000 |
| PBCV1 N-Term T4 DBD | 100 nM | T4 Buffer | A/T Blunt | 0.000 | 0.000 | 0.025 | 0.001 |
| PBCV1 N-Term T4 DBD | 100 nM | T4 Buffer | G/C Blunt | 0.000 | 0.000 | 0.019 | 0.017 |
| PBCV1 N-Term T4 DBD | 100 nM | T4 Buffer | A/T 5' SBO | 0.000 | 0.000 | 0.029 | 0.025 |
| PBCV1 N-Term T4 DBD | 100 nM | T4 Buffer | G/C 5' SBO | 0.000 | 0.000 | 0.048 | 0.021 |
| PBCV1 N-Term T4 DBD | 100 nM | T4 Buffer | A/T 3' SBO | 0.028 | 0.023 | 0.048 | 0.036 |
| PBCV1 N-Term T4 DBD | 100 nM | T4 Buffer | G/C 3' SBO | 0.000 | 0.000 | 0.199 | 0.273 |
| PBCV1 N-Term T4 DBD | 100 nM | T4 Buffer | 2BO | 0.022 | 0.011 | 0.006 | 0.010 |
| PBCV1 N-Term T4 DBD | 100 nM | T4 Buffer | 4BO | 0.818 | 0.030 | 0.003 | 0.006 |
| PBCV1 N-Term T4 DBD | 1000 nM | T4 Buffer | A/T Blunt | 0.237 | 0.008 | 0.248 | 0.001 |
| PBCV1 N-Term T4 DBD | 1000 nM | T4 Buffer | G/C Blunt | 0.175 | 0.010 | 0.271 | 0.011 |
| PBCV1 N-Term T4 DBD | 1000 nM | T4 Buffer | A/T 5' SBO | 0.008 | 0.011 | 0.041 | 0.009 |
| PBCV1 N-Term T4 DBD | 1000 nM | T4 Buffer | G/C 5' SBO | 0.045 | 0.026 | 0.076 | 0.010 |
| PBCV1 N-Term T4 DBD | 1000 nM | T4 Buffer | A/T 3' SBO | 0.327 | 0.054 | 0.323 | 0.029 |
| PBCV1 N-Term T4 DBD | 1000 nM | T4 Buffer | G/C 3' SBO | 0.305 | 0.025 | 0.580 | 0.026 |
| PBCV1 N-Term T4 DBD | 1000 nM | T4 Buffer | 2BO | 0.285 | 0.065 | 0.168 | 0.006 |
| PBCV1 N-Term T4 DBD | 1000 nM | T4 Buffer | 4BO | 0.948 | 0.005 | 0.012 | 0.001 |
| PBCV1 N-Term T4 DBD | 100 nM | QL Buffer | A/T Blunt | 0.019 | 0.001 | 0.026 | 0.004 |
| PBCV1 N-Term T4 DBD | 100 nM | QL Buffer | G/C Blunt | 0.009 | 0.001 | 0.025 | 0.020 |
| PBCV1 N-Term T4 DBD | 100 nM | QL Buffer | A/T 5' SBO | 0.000 | 0.000 | 0.019 | 0.009 |
| PBCV1 N-Term T4 DBD | 100 nM | QL Buffer | G/C 5' SBO | 0.000 | 0.000 | 0.020 | 0.014 |
| PBCV1 N-Term T4 DBD | 100 nM | QL Buffer | A/T 3' SBO | 0.066 | 0.010 | 0.072 | 0.006 |
| PBCV1 N-Term T4 DBD | 100 nM | QL Buffer | G/C 3' SBO | 0.061 | 0.008 | 0.133 | 0.007 |
| PBCV1 N-Term T4 DBD | 100 nM | QL Buffer | 2BO | 0.081 | 0.008 | 0.029 | 0.007 |
| PBCV1 N-Term T4 DBD | 100 nM | QL Buffer | 4BO | 0.918 | 0.012 | 0.012 | 0.001 |
| PBCV1 N-Term T4 DBD | 1000 nM | QL Buffer | A/T Blunt | 0.223 | 0.042 | 0.302 | 0.046 |
| PBCV1 N-Term T4 DBD | 1000 nM | QL Buffer | G/C Blunt | 0.143 | 0.029 | 0.310 | 0.065 |
| PBCV1 N-Term T4 DBD | 1000 nM | QL Buffer | A/T 5' SBO | 0.000 | 0.000 | 0.019 | 0.033 |
| PBCV1 N-Term T4 DBD | 1000 nM | QL Buffer | G/C 5' SBO | 0.030 | 0.017 | 0.089 | 0.021 |
| PBCV1 N-Term T4 DBD | 1000 nM | QL Buffer | A/T 3' SBO | 0.334 | 0.014 | 0.277 | 0.043 |
| PBCV1 N-Term T4 DBD | 1000 nM | QL Buffer | G/C 3' SBO | 0.292 | 0.009 | 0.563 | 0.031 |
| PBCV1 N-Term T4 DBD | 1000 nM | QL Buffer | 2BO | 0.599 | 0.067 | 0.158 | 0.028 |
| PBCV1 N-Term T4 DBD | 1000 nM | QL Buffer | 4BO | 0.959 | 0.018 | 0.019 | 0.021 |
| HLig3 | 100 nM | T4 Buffer | A/T Blunt | 0.388 | 0.023 | 0.025 | 0.002 |
| HLig3 | 100 nM | T4 Buffer | G/C Blunt | 0.369 | 0.048 | 0.024 | 0.006 |
| HLig3 | 100 nM | T4 Buffer | A/T 5' SBO | 0.098 | 0.013 | 0.018 | 0.002 |
| HLig3 | 100 nM | T4 Buffer | G/C 5' SBO | 0.213 | 0.034 | 0.033 | 0.007 |
| HLig3 | 100 nM | T4 Buffer | A/T 3' SBO | 0.018 | 0.005 | 0.038 | 0.010 |
| HLig3 | 100 nM | T4 Buffer | G/C 3' SBO | 0.008 | 0.007 | 0.027 | 0.008 |
| HLig3 | 100 nM | T4 Buffer | 2BO | 0.271 | 0.043 | 0.059 | 0.004 |
| HLig3 | 100 nM | T4 Buffer | 4BO | 0.833 | 0.021 | 0.000 | 0.000 |
| HLig3 | 1000 nM | T4 Buffer | A/T Blunt | 0.724 | 0.016 | 0.042 | 0.004 |
| HLig3 | 1000 nM | T4 Buffer | G/C Blunt | 0.761 | 0.009 | 0.057 | 0.002 |
| HLig3 | 1000 nM | T4 Buffer | A/T 5' SBO | 0.422 | 0.016 | 0.088 | 0.006 |
| HLig3 | 1000 nM | T4 Buffer | G/C 5' SBO | 0.480 | 0.049 | 0.029 | 0.009 |
| HLig3 | 1000 nM | T4 Buffer | A/T 3' SBO | 0.154 | 0.004 | 0.137 | 0.008 |
| HLig3 | 1000 nM | T4 Buffer | G/C 3' SBO | 0.068 | 0.009 | 0.093 | 0.003 |
| HLig3 | 1000 nM | T4 Buffer | 2BO | 0.596 | 0.023 | 0.113 | 0.008 |
| HLig3 | 1000 nM | T4 Buffer | 4BO | 0.910 | 0.046 | 0.000 | 0.000 |
| HLig3 | 100 nM | QL Buffer | A/T Blunt | 0.306 | 0.038 | 0.026 | 0.024 |
| HLig3 | 100 nM | QL Buffer | G/C Blunt | 0.414 | 0.010 | 0.022 | 0.003 |
| HLig3 | 100 nM | QL Buffer | A/T 5' SBO | 0.191 | 0.005 | 0.018 | 0.006 |
| HLig3 | 100 nM | QL Buffer | G/C 5' SBO | 0.369 | 0.084 | 0.059 | 0.049 |
| HLig3 | 100 nM | QL Buffer | A/T 3' SBO | 0.101 | 0.055 | 0.066 | 0.068 |
| HLig3 | 100 nM | QL Buffer | G/C 3' SBO | 0.000 | 0.000 | 0.025 | 0.001 |
| HLig3 | 100 nM | QL Buffer | 2BO | 0.277 | 0.047 | 0.039 | 0.010 |
| HLig3 | 100 nM | QL Buffer | 4BO | 0.802 | 0.005 | 0.000 | 0.000 |
| HLig3 | 1000 nM | QL Buffer | A/T Blunt | 0.620 | 0.063 | 0.039 | 0.038 |
| HLig3 | 1000 nM | QL Buffer | G/C Blunt | 0.751 | 0.077 | 0.095 | 0.045 |
| HLig3 | 1000 nM | QL Buffer | A/T 5' SBO | 0.684 | 0.022 | 0.072 | 0.008 |
| HLig3 | 1000 nM | QL Buffer | G/C 5' SBO | 0.613 | 0.028 | 0.033 | 0.006 |
| HLig3 | 1000 nM | QL Buffer | A/T 3' SBO | 0.331 | 0.204 | 0.217 | 0.187 |
| HLig3 | 1000 nM | QL Buffer | G/C 3' SBO | 0.044 | 0.007 | 0.128 | 0.014 |
| HLig3 | 1000 nM | QL Buffer | 2BO | 0.394 | 0.054 | 0.082 | 0.012 |
| HLig3 | 1000 nM | QL Buffer | 4BO | 0.916 | 0.017 | 0.000 | 0.000 |
| *E. coli* | 100 nM | *E. Coli* Buffer | A/T Blunt | 0.000 | 0.000 | 0.012 | 0.003 |
| *E. coli* | 100 nM | *E. Coli* Buffer | G/C Blunt | 0.000 | 0.000 | 0.018 | 0.008 |
| *E. coli* | 100 nM | *E. Coli* Buffer | A/T 5' SBO | 0.000 | 0.000 | 0.016 | 0.002 |
| *E. coli* | 100 nM | *E. Coli* Buffer | G/C 5' SBO | 0.006 | 0.002 | 0.020 | 0.003 |
| *E. coli* | 100 nM | *E. Coli* Buffer | A/T 3' SBO | 0.000 | 0.000 | 0.015 | 0.003 |
| *E. coli* | 100 nM | *E. Coli* Buffer | G/C 3' SBO | 0.000 | 0.000 | 0.010 | 0.002 |
| *E. coli* | 100 nM | *E. Coli* Buffer | 2BO | 0.008 | 0.005 | 0.013 | 0.010 |
| *E. coli* | 100 nM | *E. Coli* Buffer | 4BO | 0.427 | 0.074 | 0.000 | 0.000 |
| *E. coli* | 1000 nM | *E. Coli* Buffer | A/T Blunt | 0.000 | 0.000 | 0.009 | 0.008 |
| *E. coli* | 1000 nM | *E. Coli* Buffer | G/C Blunt | 0.002 | 0.004 | 0.009 | 0.002 |
| *E. coli* | 1000 nM | *E. Coli* Buffer | A/T 5' SBO | 0.005 | 0.005 | 0.017 | 0.005 |
| *E. coli* | 1000 nM | *E. Coli* Buffer | G/C 5' SBO | 0.056 | 0.017 | 0.012 | 0.003 |
| *E. coli* | 1000 nM | *E. Coli* Buffer | A/T 3' SBO | 0.000 | 0.000 | 0.007 | 0.005 |
| *E. coli* | 1000 nM | *E. Coli* Buffer | G/C 3' SBO | 0.000 | 0.000 | 0.004 | 0.003 |
| *E. coli* | 1000 nM | *E. Coli* Buffer | 2BO | 0.034 | 0.009 | 0.013 | 0.007 |
| *E. coli* | 1000 nM | *E. Coli* Buffer | 4BO | 0.777 | 0.007 | 0.000 | 0.000 |
| *E. coli* | 100 nM | *E. Coli* Buffer + PEG | A/T Blunt | 0.003 | 0.004 | 0.044 | 0.016 |
| *E. coli* | 100 nM | *E. Coli* Buffer + PEG | G/C Blunt | 0.000 | 0.000 | 0.008 | 0.007 |
| *E. coli* | 100 nM | *E. Coli* Buffer + PEG | A/T 5' SBO | 0.000 | 0.000 | 0.017 | 0.002 |
| *E. coli* | 100 nM | *E. Coli* Buffer + PEG | G/C 5' SBO | 0.012 | 0.010 | 0.081 | 0.114 |
| *E. coli* | 100 nM | *E. Coli* Buffer + PEG | A/T 3' SBO | 0.000 | 0.000 | 0.010 | 0.008 |
| *E. coli* | 100 nM | *E. Coli* Buffer + PEG | G/C 3' SBO | 0.000 | 0.000 | 0.013 | 0.010 |
| *E. coli* | 100 nM | *E. Coli* Buffer + PEG | 2BO | 0.019 | 0.003 | 0.020 | 0.010 |
| *E. coli* | 100 nM | *E. Coli* Buffer + PEG | 4BO | 0.702 | 0.011 | 0.000 | 0.000 |
| *E. coli* | 1000 nM | *E. Coli* Buffer + PEG | A/T Blunt | 0.000 | 0.000 | 0.059 | 0.073 |
| *E. coli* | 1000 nM | *E. Coli* Buffer + PEG | G/C Blunt | 0.000 | 0.000 | 0.012 | 0.005 |
| *E. coli* | 1000 nM | *E. Coli* Buffer + PEG | A/T 5' SBO | 0.013 | 0.003 | 0.020 | 0.001 |
| *E. coli* | 1000 nM | *E. Coli* Buffer + PEG | G/C 5' SBO | 0.246 | 0.006 | 0.017 | 0.003 |
| *E. coli* | 1000 nM | *E. Coli* Buffer + PEG | A/T 3' SBO | 0.000 | 0.000 | 0.008 | 0.001 |
| *E. coli* | 1000 nM | *E. Coli* Buffer + PEG | G/C 3' SBO | 0.002 | 0.003 | 0.009 | 0.002 |
| *E. coli* | 1000 nM | *E. Coli* Buffer + PEG | 2BO | 0.156 | 0.014 | 0.016 | 0.004 |
| *E. coli* | 1000 nM | *E. Coli* Buffer + PEG | 4BO | 0.843 | 0.003 | 0.000 | 0.000 |
| T3 | 100 nM | T4 Buffer | A/T Blunt | 0.002 | 0.003 | 0.017 | 0.004 |
| T3 | 100 nM | T4 Buffer | G/C Blunt | 0.014 | 0.003 | 0.010 | 0.005 |
| T3 | 100 nM | T4 Buffer | A/T 5' SBO | 0.000 | 0.000 | 0.018 | 0.004 |
| T3 | 100 nM | T4 Buffer | G/C 5' SBO | 0.000 | 0.000 | 0.025 | 0.016 |
| T3 | 100 nM | T4 Buffer | A/T 3' SBO | 0.000 | 0.000 | 0.013 | 0.009 |
| T3 | 100 nM | T4 Buffer | G/C 3' SBO | 0.000 | 0.000 | 0.020 | 0.008 |
| T3 | 100 nM | T4 Buffer | 2BO | 0.000 | 0.000 | 0.013 | 0.005 |
| T3 | 100 nM | T4 Buffer | 4BO | 0.867 | 0.015 | 0.000 | 0.000 |
| T3 | 1000 nM | T4 Buffer | A/T Blunt | 0.078 | 0.007 | 0.027 | 0.013 |
| T3 | 1000 nM | T4 Buffer | G/C Blunt | 0.211 | 0.078 | 0.012 | 0.003 |
| T3 | 1000 nM | T4 Buffer | A/T 5' SBO | 0.008 | 0.002 | 0.015 | 0.004 |
| T3 | 1000 nM | T4 Buffer | G/C 5' SBO | 0.016 | 0.003 | 0.014 | 0.004 |
| T3 | 1000 nM | T4 Buffer | A/T 3' SBO | 0.008 | 0.000 | 0.007 | 0.001 |
| T3 | 1000 nM | T4 Buffer | G/C 3' SBO | 0.031 | 0.004 | 0.015 | 0.011 |
| T3 | 1000 nM | T4 Buffer | 2BO | 0.000 | 0.000 | 0.013 | 0.009 |
| T3 | 1000 nM | T4 Buffer | 4BO | 0.936 | 0.001 | 0.000 | 0.000 |
| T3 | 100 nM | QL Buffer | A/T Blunt | 0.070 | 0.004 | 0.020 | 0.003 |
| T3 | 100 nM | QL Buffer | G/C Blunt | 0.154 | 0.009 | 0.012 | 0.002 |
| T3 | 100 nM | QL Buffer | A/T 5' SBO | 0.004 | 0.003 | 0.028 | 0.013 |
| T3 | 100 nM | QL Buffer | G/C 5' SBO | 0.021 | 0.000 | 0.021 | 0.004 |
| T3 | 100 nM | QL Buffer | A/T 3' SBO | 0.011 | 0.002 | 0.010 | 0.004 |
| T3 | 100 nM | QL Buffer | G/C 3' SBO | 0.028 | 0.001 | 0.013 | 0.003 |
| T3 | 100 nM | QL Buffer | 2BO | 0.000 | 0.000 | 0.012 | 0.012 |
| T3 | 100 nM | QL Buffer | 4BO | 0.919 | 0.005 | 0.000 | 0.000 |
| T3 | 1000 nM | QL Buffer | A/T Blunt | 0.333 | 0.021 | 0.028 | 0.017 |
| T3 | 1000 nM | QL Buffer | G/C Blunt | 0.564 | 0.033 | 0.012 | 0.004 |
| T3 | 1000 nM | QL Buffer | A/T 5' SBO | 0.032 | 0.006 | 0.017 | 0.002 |
| T3 | 1000 nM | QL Buffer | G/C 5' SBO | 0.130 | 0.006 | 0.015 | 0.002 |
| T3 | 1000 nM | QL Buffer | A/T 3' SBO | 0.060 | 0.006 | 0.022 | 0.005 |
| T3 | 1000 nM | QL Buffer | G/C 3' SBO | 0.146 | 0.013 | 0.038 | 0.029 |
| T3 | 1000 nM | QL Buffer | 2BO | 0.002 | 0.003 | 0.014 | 0.002 |
| T3 | 1000 nM | QL Buffer | 4BO | 0.939 | 0.001 | 0.000 | 0.000 |
| T7 | 100 nM | T4 Buffer | A/T Blunt | 0.000 | 0.000 | 0.006 | 0.010 |
| T7 | 100 nM | T4 Buffer | G/C Blunt | 0.000 | 0.000 | 0.006 | 0.011 |
| T7 | 100 nM | T4 Buffer | A/T 5' SBO | 0.000 | 0.000 | 0.021 | 0.006 |
| T7 | 100 nM | T4 Buffer | G/C 5' SBO | 0.000 | 0.000 | 0.003 | 0.006 |
| T7 | 100 nM | T4 Buffer | A/T 3' SBO | 0.000 | 0.000 | 0.017 | 0.009 |
| T7 | 100 nM | T4 Buffer | G/C 3' SBO | 0.000 | 0.000 | 0.004 | 0.006 |
| T7 | 100 nM | T4 Buffer | 2BO | 0.000 | 0.000 | 0.004 | 0.007 |
| T7 | 100 nM | T4 Buffer | 4BO | 0.781 | 0.013 | 0.000 | 0.000 |
| T7 | 1000 nM | T4 Buffer | A/T Blunt | 0.000 | 0.000 | 0.000 | 0.000 |
| T7 | 1000 nM | T4 Buffer | G/C Blunt | 0.000 | 0.000 | 0.000 | 0.000 |
| T7 | 1000 nM | T4 Buffer | A/T 5' SBO | 0.000 | 0.000 | 0.034 | 0.018 |
| T7 | 1000 nM | T4 Buffer | G/C 5' SBO | 0.000 | 0.000 | 0.000 | 0.000 |
| T7 | 1000 nM | T4 Buffer | A/T 3' SBO | 0.000 | 0.000 | 0.000 | 0.000 |
| T7 | 1000 nM | T4 Buffer | G/C 3' SBO | 0.000 | 0.000 | 0.000 | 0.000 |
| T7 | 1000 nM | T4 Buffer | 2BO | 0.000 | 0.000 | 0.013 | 0.011 |
| T7 | 1000 nM | T4 Buffer | 4BO | 0.872 | 0.012 | 0.000 | 0.000 |
| T7 | 100 nM | QL Buffer | A/T Blunt | 0.000 | 0.000 | 0.015 | 0.026 |
| T7 | 100 nM | QL Buffer | G/C Blunt | 0.000 | 0.000 | 0.000 | 0.000 |
| T7 | 100 nM | QL Buffer | A/T 5' SBO | 0.000 | 0.000 | 0.013 | 0.011 |
| T7 | 100 nM | QL Buffer | G/C 5' SBO | 0.000 | 0.000 | 0.000 | 0.000 |
| T7 | 100 nM | QL Buffer | A/T 3' SBO | 0.000 | 0.000 | 0.000 | 0.000 |
| T7 | 100 nM | QL Buffer | G/C 3' SBO | 0.000 | 0.000 | 0.005 | 0.009 |
| T7 | 100 nM | QL Buffer | 2BO | 0.000 | 0.000 | 0.013 | 0.016 |
| T7 | 100 nM | QL Buffer | 4BO | 0.893 | 0.001 | 0.000 | 0.000 |
| T7 | 1000 nM | QL Buffer | A/T Blunt | 0.000 | 0.000 | 0.000 | 0.000 |
| T7 | 1000 nM | QL Buffer | G/C Blunt | 0.000 | 0.000 | 0.000 | 0.000 |
| T7 | 1000 nM | QL Buffer | A/T 5' SBO | 0.000 | 0.000 | 0.010 | 0.009 |
| T7 | 1000 nM | QL Buffer | G/C 5' SBO | 0.000 | 0.000 | 0.000 | 0.000 |
| T7 | 1000 nM | QL Buffer | A/T 3' SBO | 0.000 | 0.000 | 0.003 | 0.003 |
| T7 | 1000 nM | QL Buffer | G/C 3' SBO | 0.000 | 0.000 | 0.007 | 0.006 |
| T7 | 1000 nM | QL Buffer | 2BO | 0.000 | 0.000 | 0.009 | 0.010 |
| T7 | 1000 nM | QL Buffer | 4BO | 0.890 | 0.002 | 0.000 | 0.000 |
| T4 | 100 nM | T4 Buffer + NaCl | A/T Blunt | 0.004 | 0.004 | 0.014 | 0.008 |
| T4 | 100 nM | T4 Buffer + NaCl | G/C Blunt | 0.000 | 0.000 | 0.018 | 0.011 |
| T4 | 100 nM | T4 Buffer + NaCl | A/T 5' SBO | 0.000 | 0.000 | 0.018 | 0.003 |
| T4 | 100 nM | T4 Buffer + NaCl | G/C 5' SBO | 0.000 | 0.000 | 0.023 | 0.004 |
| T4 | 100 nM | T4 Buffer + NaCl | A/T 3' SBO | 0.000 | 0.000 | 0.015 | 0.015 |
| T4 | 100 nM | T4 Buffer + NaCl | G/C 3' SBO | 0.000 | 0.000 | 0.008 | 0.001 |
| T4 | 100 nM | T4 Buffer + NaCl | 2BO | 0.005 | 0.002 | 0.007 | 0.002 |
| T4 | 100 nM | T4 Buffer + NaCl | 4BO | 0.857 | 0.015 | 0.000 | 0.000 |
| T4 | 1000 nM | T4 Buffer + NaCl | A/T Blunt | 0.041 | 0.013 | 0.045 | 0.028 |
| T4 | 1000 nM | T4 Buffer + NaCl | G/C Blunt | 0.002 | 0.002 | 0.008 | 0.003 |
| T4 | 1000 nM | T4 Buffer + NaCl | A/T 5' SBO | 0.000 | 0.000 | 0.018 | 0.006 |
| T4 | 1000 nM | T4 Buffer + NaCl | G/C 5' SBO | 0.000 | 0.000 | 0.013 | 0.002 |
| T4 | 1000 nM | T4 Buffer + NaCl | A/T 3' SBO | 0.000 | 0.000 | 0.008 | 0.005 |
| T4 | 1000 nM | T4 Buffer + NaCl | G/C 3' SBO | 0.000 | 0.000 | 0.007 | 0.001 |
| T4 | 1000 nM | T4 Buffer + NaCl | 2BO | 0.046 | 0.012 | 0.014 | 0.004 |
| T4 | 1000 nM | T4 Buffer + NaCl | 4BO | 0.901 | 0.007 | 0.000 | 0.000 |
| T4 | 100 nM | QL Buffer + NaCl | A/T Blunt | 0.023 | 0.005 | 0.020 | 0.002 |
| T4 | 100 nM | QL Buffer + NaCl | G/C Blunt | 0.001 | 0.002 | 0.009 | 0.002 |
| T4 | 100 nM | QL Buffer + NaCl | A/T 5' SBO | 0.000 | 0.000 | 0.016 | 0.004 |
| T4 | 100 nM | QL Buffer + NaCl | G/C 5' SBO | 0.000 | 0.000 | 0.015 | 0.003 |
| T4 | 100 nM | QL Buffer + NaCl | A/T 3' SBO | 0.000 | 0.000 | 0.005 | 0.002 |
| T4 | 100 nM | QL Buffer + NaCl | G/C 3' SBO | 0.000 | 0.000 | 0.009 | 0.002 |
| T4 | 100 nM | QL Buffer + NaCl | 2BO | 0.030 | 0.001 | 0.011 | 0.004 |
| T4 | 100 nM | QL Buffer + NaCl | 4BO | 0.889 | 0.005 | 0.000 | 0.000 |
| T4 | 1000 nM | QL Buffer + NaCl | A/T Blunt | 0.186 | 0.038 | 0.018 | 0.003 |
| T4 | 1000 nM | QL Buffer + NaCl | G/C Blunt | 0.021 | 0.011 | 0.025 | 0.011 |
| T4 | 1000 nM | QL Buffer + NaCl | A/T 5' SBO | 0.001 | 0.002 | 0.015 | 0.007 |
| T4 | 1000 nM | QL Buffer + NaCl | G/C 5' SBO | 0.005 | 0.001 | 0.026 | 0.034 |
| T4 | 1000 nM | QL Buffer + NaCl | A/T 3' SBO | 0.005 | 0.001 | 0.021 | 0.006 |
| T4 | 1000 nM | QL Buffer + NaCl | G/C 3' SBO | 0.003 | 0.002 | 0.017 | 0.010 |
| T4 | 1000 nM | QL Buffer + NaCl | 2BO | 0.318 | 0.018 | 0.014 | 0.004 |
| T4 | 1000 nM | QL Buffer + NaCl | 4BO | 0.908 | 0.012 | 0.000 | 0.000 |
| PBCV1 Sso7d in Trans | 100 nM | T4 Buffer | A/T Blunt | 0.000 | 0.000 | 0.012 | 0.003 |
| PBCV1 Sso7d in Trans | 100 nM | T4 Buffer | G/C Blunt | 0.008 | 0.001 | 0.015 | 0.006 |
| PBCV1 Sso7d in Trans | 100 nM | T4 Buffer | A/T 5' SBO | 0.000 | 0.000 | 0.021 | 0.001 |
| PBCV1 Sso7d in Trans | 100 nM | T4 Buffer | G/C 5' SBO | 0.000 | 0.000 | 0.009 | 0.010 |
| PBCV1 Sso7d in Trans | 100 nM | T4 Buffer | A/T 3' SBO | 0.025 | 0.008 | 0.034 | 0.013 |
| PBCV1 Sso7d in Trans | 100 nM | T4 Buffer | G/C 3' SBO | 0.024 | 0.006 | 0.042 | 0.010 |
| PBCV1 Sso7d in Trans | 100 nM | T4 Buffer | 2BO | 0.028 | 0.003 | 0.022 | 0.016 |
| PBCV1 Sso7d in Trans | 100 nM | T4 Buffer | 4BO | 0.913 | 0.002 | 0.000 | 0.000 |
| PBCV1 Sso7d in Trans | 1000 nM | T4 Buffer | A/T Blunt | 0.000 | 0.000 | 0.034 | 0.017 |
| PBCV1 Sso7d in Trans | 1000 nM | T4 Buffer | G/C Blunt | 0.000 | 0.000 | 0.006 | 0.003 |
| PBCV1 Sso7d in Trans | 1000 nM | T4 Buffer | A/T 5' SBO | 0.000 | 0.000 | 0.016 | 0.014 |
| PBCV1 Sso7d in Trans | 1000 nM | T4 Buffer | G/C 5' SBO | 0.000 | 0.000 | 0.020 | 0.017 |
| PBCV1 Sso7d in Trans | 1000 nM | T4 Buffer | A/T 3' SBO | 0.053 | 0.007 | 0.044 | 0.000 |
| PBCV1 Sso7d in Trans | 1000 nM | T4 Buffer | G/C 3' SBO | 0.031 | 0.005 | 0.065 | 0.003 |
| PBCV1 Sso7d in Trans | 1000 nM | T4 Buffer | 2BO | 0.068 | 0.004 | 0.073 | 0.015 |
| PBCV1 Sso7d in Trans | 1000 nM | T4 Buffer | 4BO | 0.912 | 0.009 | 0.000 | 0.000 |
| PBCV1 Sso7d in Trans | 100 nM | QL Buffer | A/T Blunt | 0.000 | 0.000 | 0.030 | 0.021 |
| PBCV1 Sso7d in Trans | 100 nM | QL Buffer | G/C Blunt | 0.000 | 0.000 | 0.005 | 0.005 |
| PBCV1 Sso7d in Trans | 100 nM | QL Buffer | A/T 5' SBO | 0.002 | 0.003 | 0.022 | 0.027 |
| PBCV1 Sso7d in Trans | 100 nM | QL Buffer | G/C 5' SBO | 0.000 | 0.000 | 0.014 | 0.024 |
| PBCV1 Sso7d in Trans | 100 nM | QL Buffer | A/T 3' SBO | 0.120 | 0.010 | 0.045 | 0.012 |
| PBCV1 Sso7d in Trans | 100 nM | QL Buffer | G/C 3' SBO | 0.087 | 0.007 | 0.079 | 0.008 |
| PBCV1 Sso7d in Trans | 100 nM | QL Buffer | 2BO | 0.081 | 0.012 | 0.037 | 0.010 |
| PBCV1 Sso7d in Trans | 100 nM | QL Buffer | 4BO | 0.912 | 0.002 | 0.000 | 0.000 |
| PBCV1 Sso7d in Trans | 1000 nM | QL Buffer | A/T Blunt | 0.055 | 0.059 | 0.036 | 0.008 |
| PBCV1 Sso7d in Trans | 1000 nM | QL Buffer | G/C Blunt | 0.009 | 0.008 | 0.027 | 0.009 |
| PBCV1 Sso7d in Trans | 1000 nM | QL Buffer | A/T 5' SBO | 0.000 | 0.000 | 0.023 | 0.007 |
| PBCV1 Sso7d in Trans | 1000 nM | QL Buffer | G/C 5' SBO | 0.000 | 0.000 | 0.023 | 0.008 |
| PBCV1 Sso7d in Trans | 1000 nM | QL Buffer | A/T 3' SBO | 0.223 | 0.012 | 0.079 | 0.012 |
| PBCV1 Sso7d in Trans | 1000 nM | QL Buffer | G/C 3' SBO | 0.200 | 0.040 | 0.146 | 0.022 |
| PBCV1 Sso7d in Trans | 1000 nM | QL Buffer | 2BO | 0.192 | 0.039 | 0.103 | 0.013 |
| PBCV1 Sso7d in Trans | 1000 nM | QL Buffer | 4BO | 0.867 | 0.009 | 0.000 | 0.000 |
| T3 | 100 nM | T4 Buffer + NaCl | A/T Blunt | 0.000 | 0.000 | 0.005 | 0.008 |
| T3 | 100 nM | T4 Buffer + NaCl | G/C Blunt | 0.000 | 0.000 | 0.000 | 0.000 |
| T3 | 100 nM | T4 Buffer + NaCl | A/T 5' SBO | 0.000 | 0.000 | 0.004 | 0.007 |
| T3 | 100 nM | T4 Buffer + NaCl | G/C 5' SBO | 0.000 | 0.000 | 0.036 | 0.048 |
| T3 | 100 nM | T4 Buffer + NaCl | A/T 3' SBO | 0.000 | 0.000 | 0.012 | 0.010 |
| T3 | 100 nM | T4 Buffer + NaCl | G/C 3' SBO | 0.000 | 0.000 | 0.010 | 0.006 |
| T3 | 100 nM | T4 Buffer + NaCl | 2BO | 0.000 | 0.000 | 0.016 | 0.022 |
| T3 | 100 nM | T4 Buffer + NaCl | 4BO | 0.854 | 0.008 | 0.000 | 0.000 |
| T3 | 1000 nM | T4 Buffer + NaCl | A/T Blunt | 0.000 | 0.000 | 0.013 | 0.011 |
| T3 | 1000 nM | T4 Buffer + NaCl | G/C Blunt | 0.000 | 0.000 | 0.010 | 0.009 |
| T3 | 1000 nM | T4 Buffer + NaCl | A/T 5' SBO | 0.000 | 0.000 | 0.001 | 0.002 |
| T3 | 1000 nM | T4 Buffer + NaCl | G/C 5' SBO | 0.000 | 0.000 | 0.000 | 0.000 |
| T3 | 1000 nM | T4 Buffer + NaCl | A/T 3' SBO | 0.000 | 0.000 | 0.002 | 0.004 |
| T3 | 1000 nM | T4 Buffer + NaCl | G/C 3' SBO | 0.000 | 0.000 | 0.007 | 0.005 |
| T3 | 1000 nM | T4 Buffer + NaCl | 2BO | 0.000 | 0.000 | 0.048 | 0.017 |
| T3 | 1000 nM | T4 Buffer + NaCl | 4BO | 0.910 | 0.003 | 0.000 | 0.000 |
| T3 | 100 nM | QL Buffer + NaCl | A/T Blunt | 0.001 | 0.001 | 0.008 | 0.007 |
| T3 | 100 nM | QL Buffer + NaCl | G/C Blunt | 0.000 | 0.000 | 0.006 | 0.008 |
| T3 | 100 nM | QL Buffer + NaCl | A/T 5' SBO | 0.000 | 0.000 | 0.001 | 0.002 |
| T3 | 100 nM | QL Buffer + NaCl | G/C 5' SBO | 0.000 | 0.000 | 0.004 | 0.006 |
| T3 | 100 nM | QL Buffer + NaCl | A/T 3' SBO | 0.026 | 0.002 | 0.017 | 0.004 |
| T3 | 100 nM | QL Buffer + NaCl | G/C 3' SBO | 0.021 | 0.007 | 0.041 | 0.004 |
| T3 | 100 nM | QL Buffer + NaCl | 2BO | 0.017 | 0.004 | 0.023 | 0.010 |
| T3 | 100 nM | QL Buffer + NaCl | 4BO | 0.913 | 0.006 | 0.000 | 0.000 |
| T3 | 1000 nM | QL Buffer + NaCl | A/T Blunt | 0.018 | 0.003 | 0.031 | 0.006 |
| T3 | 1000 nM | QL Buffer + NaCl | G/C Blunt | 0.007 | 0.007 | 0.029 | 0.006 |
| T3 | 1000 nM | QL Buffer + NaCl | A/T 5' SBO | 0.000 | 0.000 | 0.000 | 0.000 |
| T3 | 1000 nM | QL Buffer + NaCl | G/C 5' SBO | 0.000 | 0.000 | 0.000 | 0.000 |
| T3 | 1000 nM | QL Buffer + NaCl | A/T 3' SBO | 0.123 | 0.036 | 0.115 | 0.029 |
| T3 | 1000 nM | QL Buffer + NaCl | G/C 3' SBO | 0.082 | 0.013 | 0.186 | 0.035 |
| T3 | 1000 nM | QL Buffer + NaCl | 2BO | 0.110 | 0.008 | 0.082 | 0.003 |
| T3 | 1000 nM | QL Buffer + NaCl | 4BO | 0.928 | 0.005 | 0.000 | 0.000 |
| HLig3 | 100 nM | T4 Buffer + NaCl | A/T Blunt | 0.009 | 0.003 | 0.015 | 0.005 |
| HLig3 | 100 nM | T4 Buffer + NaCl | G/C Blunt | 0.000 | 0.000 | 0.012 | 0.005 |
| HLig3 | 100 nM | T4 Buffer + NaCl | A/T 5' SBO | 0.000 | 0.000 | 0.020 | 0.003 |
| HLig3 | 100 nM | T4 Buffer + NaCl | G/C 5' SBO | 0.000 | 0.000 | 0.020 | 0.008 |
| HLig3 | 100 nM | T4 Buffer + NaCl | A/T 3' SBO | 0.000 | 0.000 | 0.014 | 0.003 |
| HLig3 | 100 nM | T4 Buffer + NaCl | G/C 3' SBO | 0.000 | 0.000 | 0.012 | 0.006 |
| HLig3 | 100 nM | T4 Buffer + NaCl | 2BO | 0.000 | 0.000 | 0.012 | 0.005 |
| HLig3 | 100 nM | T4 Buffer + NaCl | 4BO | 0.834 | 0.022 | 0.000 | 0.000 |
| HLig3 | 1000 nM | T4 Buffer + NaCl | A/T Blunt | 0.120 | 0.130 | 0.023 | 0.011 |
| HLig3 | 1000 nM | T4 Buffer + NaCl | G/C Blunt | 0.009 | 0.002 | 0.009 | 0.002 |
| HLig3 | 1000 nM | T4 Buffer + NaCl | A/T 5' SBO | 0.002 | 0.002 | 0.016 | 0.002 |
| HLig3 | 1000 nM | T4 Buffer + NaCl | G/C 5' SBO | 0.027 | 0.008 | 0.015 | 0.004 |
| HLig3 | 1000 nM | T4 Buffer + NaCl | A/T 3' SBO | 0.000 | 0.000 | 0.005 | 0.004 |
| HLig3 | 1000 nM | T4 Buffer + NaCl | G/C 3' SBO | 0.000 | 0.000 | 0.006 | 0.005 |
| HLig3 | 1000 nM | T4 Buffer + NaCl | 2BO | 0.003 | 0.006 | 0.017 | 0.009 |
| HLig3 | 1000 nM | T4 Buffer + NaCl | 4BO | 0.878 | 0.020 | 0.000 | 0.000 |
| HLig3 | 100 nM | QL Buffer + NaCl | A/T Blunt | 0.041 | 0.006 | 0.019 | 0.006 |
| HLig3 | 100 nM | QL Buffer + NaCl | G/C Blunt | 0.004 | 0.004 | 0.007 | 0.006 |
| HLig3 | 100 nM | QL Buffer + NaCl | A/T 5' SBO | 0.006 | 0.001 | 0.016 | 0.002 |
| HLig3 | 100 nM | QL Buffer + NaCl | G/C 5' SBO | 0.039 | 0.017 | 0.011 | 0.003 |
| HLig3 | 100 nM | QL Buffer + NaCl | A/T 3' SBO | 0.000 | 0.000 | 0.004 | 0.001 |
| HLig3 | 100 nM | QL Buffer + NaCl | G/C 3' SBO | 0.000 | 0.000 | 0.006 | 0.002 |
| HLig3 | 100 nM | QL Buffer + NaCl | 2BO | 0.006 | 0.005 | 0.021 | 0.019 |
| HLig3 | 100 nM | QL Buffer + NaCl | 4BO | 0.857 | 0.001 | 0.000 | 0.000 |
| HLig3 | 1000 nM | QL Buffer + NaCl | A/T Blunt | 0.226 | 0.028 | 0.025 | 0.003 |
| HLig3 | 1000 nM | QL Buffer + NaCl | G/C Blunt | 0.204 | 0.028 | 0.085 | 0.006 |
| HLig3 | 1000 nM | QL Buffer + NaCl | A/T 5' SBO | 0.119 | 0.011 | 0.043 | 0.005 |
| HLig3 | 1000 nM | QL Buffer + NaCl | G/C 5' SBO | 0.355 | 0.031 | 0.013 | 0.003 |
| HLig3 | 1000 nM | QL Buffer + NaCl | A/T 3' SBO | 0.042 | 0.014 | 0.029 | 0.019 |
| HLig3 | 1000 nM | QL Buffer + NaCl | G/C 3' SBO | 0.032 | 0.013 | 0.013 | 0.006 |
| HLig3 | 1000 nM | QL Buffer + NaCl | 2BO | 0.087 | 0.002 | 0.128 | 0.014 |
| HLig3 | 1000 nM | QL Buffer + NaCl | 4BO | 0.872 | 0.011 | 0.000 | 0.000 |
